# Weighted Frequent Gene Co-expression Network Mining to Identify Genes Involved in Genome Stability

**DOI:** 10.1371/journal.pcbi.1002656

**Published:** 2012-08-30

**Authors:** Jie Zhang, Kewei Lu, Yang Xiang, Muhtadi Islam, Shweta Kotian, Zeina Kais, Cindy Lee, Mansi Arora, Hui-wen Liu, Jeffrey D. Parvin, Kun Huang

**Affiliations:** 1 Department of Biomedical Informatics, The Ohio State University, Columbus, Ohio, United States of America; 2 Comprehensive Cancer Center, Biomedical Informatics Shared Resource, The Ohio State University, Columbus, Ohio, United States of America; 3 Department of Computer Sciences and Engineering, The Ohio State University, Columbus, Ohio, United States of America; University of Chicago, United States of America

## Abstract

Gene co-expression network analysis is an effective method for predicting gene functions and disease biomarkers. However, few studies have systematically identified co-expressed genes involved in the molecular origin and development of various types of tumors. In this study, we used a network mining algorithm to identify tightly connected gene co-expression networks that are frequently present in microarray datasets from 33 types of cancer which were derived from 16 organs/tissues. We compared the results with networks found in multiple normal tissue types and discovered 18 tightly connected frequent networks in cancers, with highly enriched functions on cancer-related activities. Most networks identified also formed physically interacting networks. In contrast, only 6 networks were found in normal tissues, which were highly enriched for housekeeping functions. The largest cancer network contained many genes with genome stability maintenance functions. We tested 13 selected genes from this network for their involvement in genome maintenance using two cell-based assays. Among them, 10 were shown to be involved in either homology-directed DNA repair or centrosome duplication control including the well- known cancer marker MKI67. Our results suggest that the commonly recognized characteristics of cancers are supported by highly coordinated transcriptomic activities. This study also demonstrated that the co-expression network directed approach provides a powerful tool for understanding cancer physiology, predicting new gene functions, as well as providing new target candidates for cancer therapeutics.

## Author Summary

Proteins interact with each other in a network manner to precisely regulate complicated physiological functions of life. Diseases such as cancer may occur if the network regulations go wrong. In cancer research, network mining has been utilized to identify biomarkers, predict therapeutic targets, and discover new mechanisms for cancer development. Among these applications, the search for genes with similar expression patterns (co-expression) over different samples is particularly successful. However, few network mining approaches were systematically applied to different types of cancers to extract common cancer features. We carried out a systematic study to identify frequently co-expressed gene networks in multiple cancers and compared them with the gene networks found in multiple normal tissues. We found dramatic differences between networks from the two sources, with gene networks in cancer corresponding to specific traits of cancer. Specifically, the largest gene network in cancer contains many genes with cell cycle control and DNA stability functions. We thus predicted that a set of poorly studied genes in this network share similar functions and validated that most of these genes are involved in DNA break repair or proper cell division. To the best of our knowledge, this is the largest scale of such a study.

## Introduction

Distinct types of human cancer share similar traits, including rapid cell proliferation, loss of cell identity, and the ability to migrate and seed malignant tumors in distal locations. Understanding these common traits and identifying the underlying genes/networks are key to gaining insight into cancer physiology, and, ultimately, to prevent and cure cancer. With cancer gene expression microarray datasets increasingly accumulated in central repositories, many bioinformatics data analysis methods have been developed to identify cancer related genes, characterize cancer subtypes and discover gene signatures for prognosis and treatment prediction. As an example, in breast cancer research, a supervised approach was adopted to select 70 genes as biomarkers for breast cancer prognosis [1], [2] and was successfully tested in clinical settings [3]. However, a major drawback of such an approach is that the selected gene features are usually not functionally related and hence, cannot reveal key biological mechanisms and processes behind different patient groups.

In order to overcome this hurdle to identify functionally related genes associated with disease development and prognosis, several approaches have been adopted. One such approach is gene co-expression analysis, which identifies groups of genes that are highly correlated in expression levels across multiple samples [4]–[9]. The metric to measure the correlation is usually the correlation coefficient (e.g., Pearson correlation coefficient or PCC) between expression profiles of two genes [4], [5], [10]. Using this approach, we were able to identify new gene functions in regulating cell mitosis in breast cancer [5], [11] by studying genes that have high correlation with the expression of the DNA repair protein, BRCA1.

By applying an advanced network mining algorithm, dense modules of highly co-expressed genes can be identified which can lead to the discovery of new gene functions, disease genes and biomarkers. For example, Horvath's group has developed a series of weighted gene co-expression network analyses using a hierarchical clustering based approach [6], [10], [12]–[15]. This method was applied to identify disease-associated genes such as *ASPM* in glioblastoma [7].

**Figure 1:**
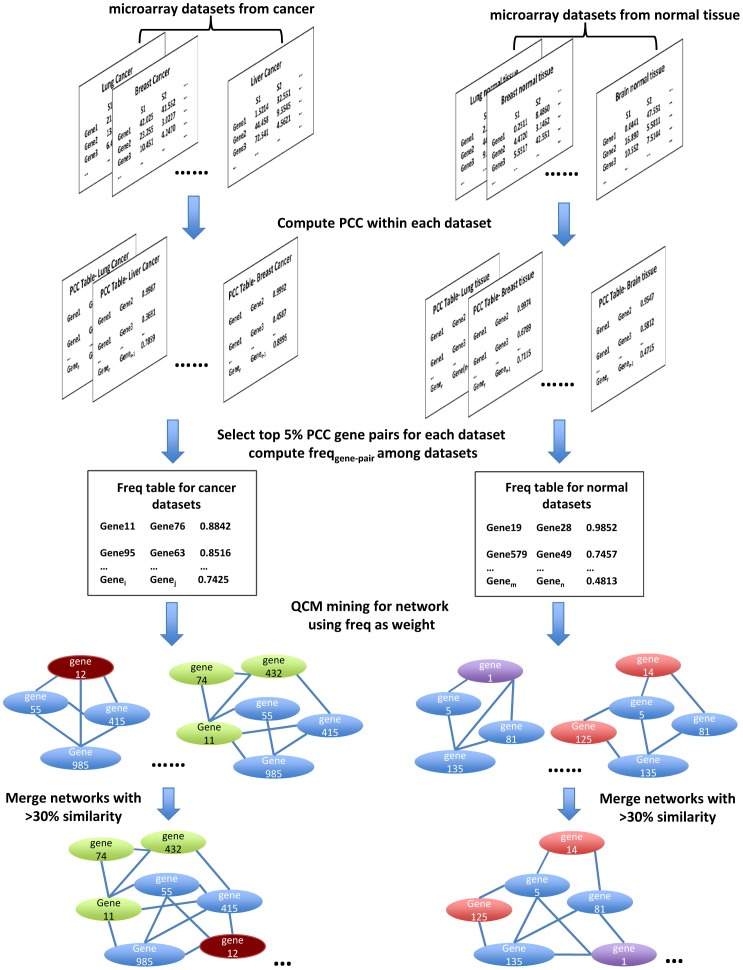
Workflow to mine frequent co-expression network using QCM from cancer and normal tissue microarray datasets. Blue ovals indicate gene members shared by different networks, ovals in other colors indicate genes unique to each network.

In this study, we hypothesize that studying clusters of frequently co-expressed genes in multiple types of cancers can shed light on the gene expression regulatory basis for common traits in cancer. We developed a workflow to test this hypothesis (Figure 1), and implemented a state-of-the-art weighted network mining algorithm called QCM (Quasi-Clique Merger [16]) to identify the gene co-expression clusters from the common cancer background using gene expression data from multiple types of cancers. Then, we further predicted the gene functions based on the networks we identified and their GO-term enrichment analysis, and validated our prediction using cell-based assays.

The QCM algorithm mines dense sub-network components in a weighted network. In contrast to traditional quasi-clique mining algorithms [4], [17], [18], QCM fully utilizes the weight of edges without turning them into un-weighted edges by a threshold cutoff. In addition, QCM returns dense sub-network components that allow overlaps of both vertices and edges. This feature makes it more appealing for mining biological networks than clustering algorithms. Thirdly and most importantly, QCM was proven mathematically to be able to generate high density sub-networks [16], which correspond to tightly co-expressed clusters of genes in our study.

Gene signatures or networks have been identified as predictive/prognostic biomarkers based on certain cancer type microarray data. However, few studies have been applied to identify cancer associated genes and therapeutic targets in multiple cancer types at the level of the functional gene module, in which gene clusters are functionally and possibly physically interacting with each other. It has been demonstrated by analyzing 507 co-expression modules and 665 gene signatures that co-expression network mining is a powerful tool to search for functional enriched modules [19]. Instead of using differential gene expression analysis, our approach is to directly mine frequent gene networks that are present in large- scale datasets of multiple cancer types, and compare them with those found in normal tissues to understand the pathways and networks that cause the major difference between cancer and normal tissue. In addition, it was reported that previous co-expression network searches often resulted in non-reproducible or poorly overlapped gene signatures/networks [20], which may have been due to arbitrary thresholds, results sensitive to parameter tuning, lack of generality or the lack of biological validation of the gene functions and interactions. We attempted to solve these problems by applying a weighted network mining algorithm to identify frequently presented co-expression gene networks on a common cancer background, and then further validated the findings with biological experimental evidence.

## Results

### Identification of high frequency co-expression networks in cancer and normal samples

Our workflow to identify tightly clustered frequent co-expression networks was developed as follows (Figure 1): First, we selected a large number of gene expression datasets for 33 different types of cancers (originated from 16 tissue types, Table 1), including sarcoma, carcinoma, adenocarcinoma, leukemia, lymphoma, and brain cancer. As a comparison, we selected microarray datasets from nine different normal tissues. The datasets were selected such that the sample size in each dataset is above a minimal threshold to maintain the significance level of PCC computation (p-values<0.05 for PCC values larger than the threshold as described in Materials and Methods). In this study, all the selected datasets have at least 30 samples, which is comparable to other co-expression network studies [14], [21]. To avoid systematic bias between different microarray platforms, we further restricted our datasets to a single platform. All the selected datasets were generated using Affymetrix HU133 Plus 2.0 Genechip. Next, a total of 2.17×10^8^ (20,827×20,826/2) gene-pair expression correlations (PCC) were computed within each dataset, and a frequency table was built for identified gene pairs with high correlation between their expression profiles in each dataset. The frequencies of highly correlated gene pairs were then used as weights to build a weighted gene co-expression frequency network (WGCFN). Third, we implemented QCM to identify high frequency gene co-expression networks in multiple types of cancers from the WGCFN for cancers and compared them to those identified in multiple types of normal tissues. In the final step, identified networks with similar members (overlaps above 30%) were merged iteratively to generate the final networks. This workflow runs parallel for the datasets from multiple cancer types and from multiple normal tissues.

**Table 1:** Summary of the sample information for microarray datasets used in the study.

GSE NO.	Cancer Type	Sample Size	Comments
GSE22138	uveal melanoma	63	
GSE12460	neuroblastoma	64	
GSE23980	soft tissue sarcoma	171	
GSE18864	breast cancer all types	84	
GSE17920	Hodgkin Lymphoma	130	
GSE19069	T-cell lymphoma	137	exclude 10 T-cell controls
GSE17951	prostate cancer	154	
GSE16237	neuroblastoma	51	
GSE10445	lung adenocarcinoma, large cell carcinoma	72	
GSE11151	9 types of renal cancer	62	exclude 5 normal samples
GSE4290	astrocytomas, oligodendrogliomas and glioblastomas.	180	exclude 23 non-tumor samples
GSE10327	medulloblastoma	62	
GSE3141	lung cancer	111	
GSE16515	pancreatic cancer	36	exclude normal samples
GSE18842	non-small cell lung cancer	46	45 controls need remove
GSE10245	non-small cell lung cancer	58	
GSE9829	hepatocellular carcinoma	194	
GSE9891	ovarian tumor	285	
GSE10358	acute myeloid leukemia	188	
GSE10846	diffuse large B cell lymphoma	414	drug treated
GSE11877	pediatric acute lymphathetic leukemia	207	
GSE13041	glioblastomas	267	
GSE14333	colorectal cancer	290	
GSE15459	gastric tumor	200	
GSE16382	soft tissue sarcoma	183	
GSE21653	medullary breast cancers	266	
GSE21687	ependymoma	83	

Normal tissue datasets			
GSE NO.	Tissue Type	Sample Size	Comments
GSE18842	non-small cell lung cancer patient normal tissue	45	exclude cancer samples
GSE17913	non-smoker oral mucosa	40	exclude smoker samples
GSE8671	normal colon mucosa	32	exclude cancer samples
GSE1643	normal lung tissues	40	
GSE21138	prefrontal cortex	30	exclude Schizophrenia samples
GSE13564	prefrontal cortex	44	
GSE7307	90 types of tissues	677	Exclude disease samples, get 23 endometrium and 22 myometrium samples
GSE11882	brain sample	173	used 43 samples of hippocampus tissue sample

The algorithm identified 111 gene co-expression networks (average network density 0.81±0.05) from cancer tissue gene expression microarray datasets before the merging step, and 70 networks for normal tissues (average network density 0.73±0.04) before the merging step. As a comparison, the average network density of 1000 randomly selected gene subsets (regardless of the subset size) was much lower as expected, and was close to the density of the overall network (0.0497, based on 20,827 genes).

We merged the networks with at least 30% similarity, obtained 18 distinctive networks in cancer datasets, and 6 networks in normal tissue datasets (Table 2, Table S1, Table S2). Despite the high diversity of cancer types, GO term enrichment analysis showed that the networks found from cancer datasets are highly enriched in elevated activities specific to cancer cells, such as *cell proliferation, immune response, and cancer microenvironment construction*, while the normal tissue networks are generally involved in housekeeping functions such as *cell respiration*, *metabolism*, and *protein synthesis*. For the networks that share similar GO term enrichment between cancer and normal tissue datasets, the cancer network generally includes most of the members of the normal tissue network but also contains many more genes. This indicates that the housekeeping functions in the cancer cell may exceed its normal range, allowing it to become more interconnected with other biological processes and pathways, which may contribute to the excessive uncontrollable growth of cancer cells.

**Table 2:** Summary of co-expression networks identified from multiple cancer datasets vs. normal tissue datasets.

	Networks from cancer Datasets			Networks from normal tissue datasets		
Network ID	Network size in merged network	Top biological processes in the merged network	p-value	Network size in merged network	Top biological processes in the merged network	p-value
1	412	Mitotic cell cycle	6.30E-130	198	Cellular respiration	5.31E-72
2	260	Immune response	1.67E-57	71	Protein synthesis	2.84E-99
3	136	Protein synthesis	1.36E-138	60	No significantly enriched BP	
4	73	Cell cycle; Cell-to-cell communication; connective tissue development	2.41E-03	25	Protein synthesis	4.09E-49
5	61	Type I interferon mediated signaling	8.42E-37	15	Mitotic cell cycle	2.52E-8
6	57	Extracellular matrix organization	1.73E-22	11	Immune response	2.80E-5
7	45	Humoral immune response	1.07E-19			
8	36	Immune response	2.74E-17			
9	27	No significantly enriched BP	n.a.			
10	22	Antigen processing and presentation	2.38E-38			
11	20	Antigen processing and presentation via MHC class II	5.28E-35			
12	12	Blood vessel development	1.40E-2			
13	11	Protein synthesis	8.04E-4			
14	11	Respiratory electron transport chain	7.37E-5			
15	11	No significantly enriched BP	n.a.			
16	10	RNA processing	2.68E-3			
17	10	No significantly enriched BP	n.a.			
18	10	Cellular respiration	1.80E-16			

As a comparison and the test for our QC*M* network mining workflow, we also applied the workflow described above (Figure 1) to the lung cancer samples of a single dataset (GSE18842, 46 samples), then to the normal lung tissue of the same dataset (45 samples). Similar observations from multiple cancer types vs. multiple normal tissue types also hold for the network mining results from single cancer type and the matching normal tissue. There are more and denser networks identified from lung cancer samples as compared with those from normal lung tissue. For the networks identified from lung cancer samples, they are enriched with functions related to cancer cells, such as *DNA mismatch repair*, *immune response*, and *extracellular matrix (ECM) construction and organization* (Table S4), whereas the networks from normal lung tissue are instead enriched with housekeeping functions such as *protein synthesis*, *cell metabolism*, and *microtubule-based activity* (Table S5). We also identified several immune response clusters from the normal lung tissue, presumably due to the fact that these normal lung tissue samples were obtained from the lung cancer patients and as a result, immune response signals induced from lung cancer can be spread to neighbor normal lung tissue. From this example, we conclude that the observations we have made in aggregate were also true in specific examples.

**Figure 2:**
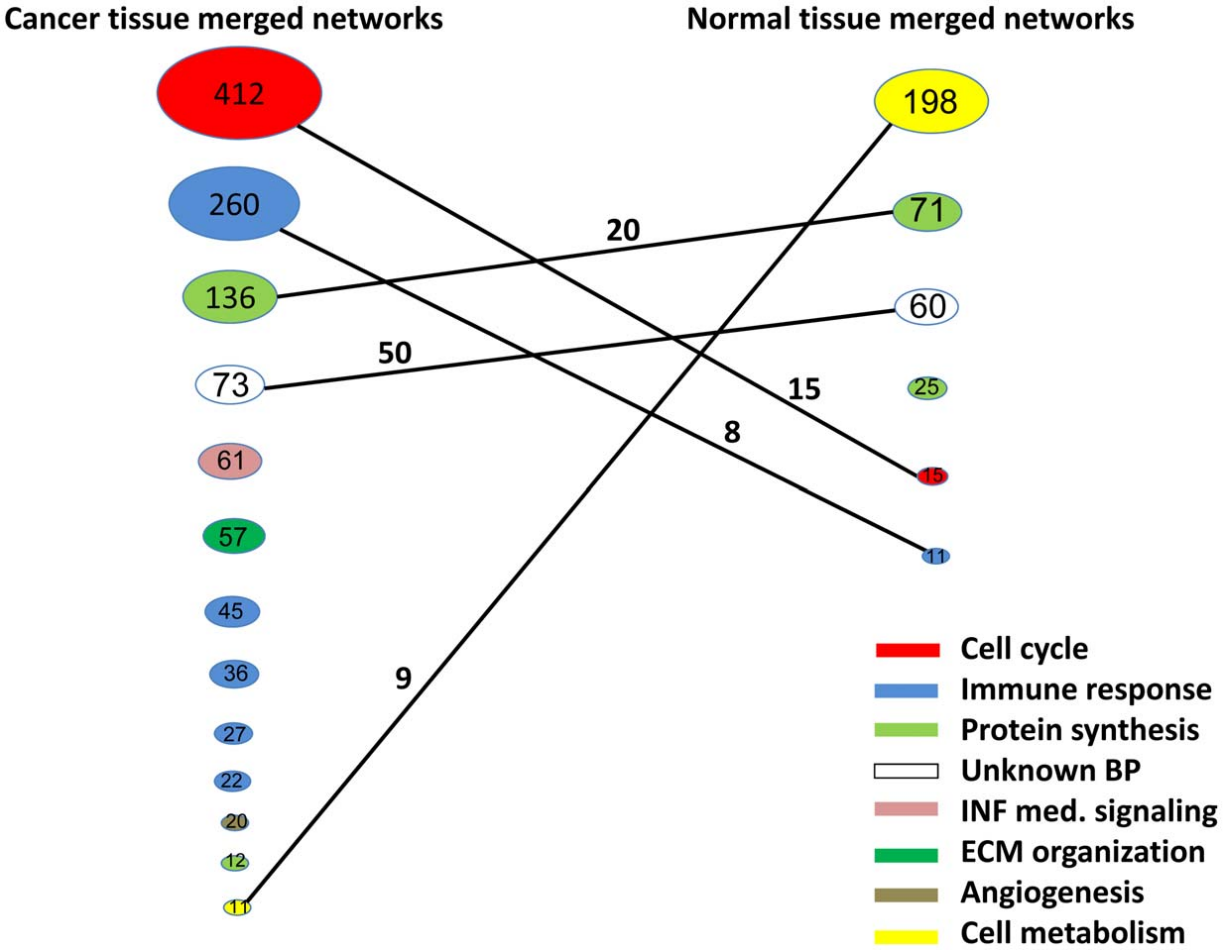
Comparison of networks identified from multiple cancer vs. normal tissue microarray datasets. Top 13 networks (ranked by size) were shown. The size of each circle represents the relative size of each network. The numbers inside the circles indicate the size of the network. The numbers above the connection line indicate the numbers of common genes shared by the two networks. Different top-enriched biological functions in each network were assigned with different colors. ECM: extracellular matrix construction. Parameter settings are: β = 0.8, γ = 0.8, λ = 2.0, t = 1.0 (for networks from cancer datasets); β = 0.8, γ = 0.7, λ = 2.0, t = 1.0 (for networks from normal tissue datasets).

**Figure 3:**
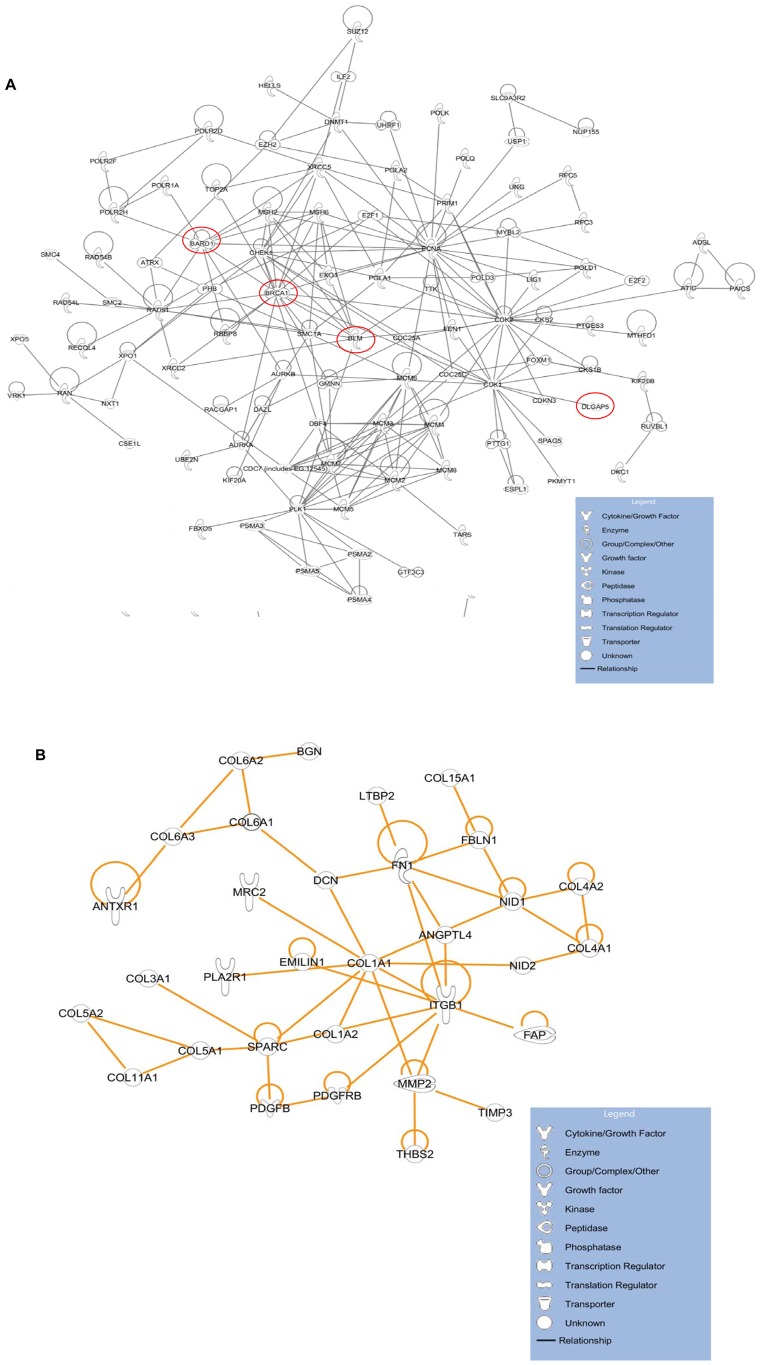
Validated protein-protein interactions on genes from networks identified from cancer datasets using IPA. The edges represent validated protein-protein interactions obtained from Ingenuity Knowledge Base. The nodes are gene members. Only members with connection to other members are shown. A: Validated protein-protein interactions on genes from Cancer Network 1 (cell proliferation/cell cycle control network) using IPA. The red circles indicate the genes further selected for genome stability function assays using RNAi. B: Validated protein-protein interactions on genes from Cancer Network 6 (extracellular matrix network) using IPA.

Network 1 is the predominant network identified consistently from cancer datasets regardless of the parameter setting (Figure 2, Table 2, Table S1, Table S3). By contrast, only a small portion of this network with looser connections was found from normal tissues (Figure 2, Table S2). Network 1 includes most of the genes that are frequently identified in a variety of gene signatures studies of the cancer microarray (Table 3, Table S1) [9], [22]–[28], and contains some less studied genes as well. The genes in this network are highly enriched in cell proliferation and genome stability maintenance functions such as *cell cycle control/regulation*, *mitotic division*, and *DNA damage response (DDR)*. After querying the Ingenuity Knowledge Base for experimentally validated protein-protein interactions, we found that 99 out of the 412 gene products from Network 1 are connected to form a tight protein-protein interaction (PPI) network, as shown in Figure 3A (enrichment p- value 5.937E-217). Similarly, 33 out of the 57 genes from Network 6 are connected in a dense PPI network (enrichment p-value 1.564E-52, Figure 3B), which is involved in an extracellular matrix formation. In addition, we also tested this using a different PPI dataset obtained from the Protein Interaction Network Analysis platform (PINA). Null distributions were generated from repetitive 500 random selections of the same number of genes as networks 1 or 6 in PPI interaction database PINA. Next, z-scores of PPI hits in networks 1 and 6 were obtained from each distribution as described in the Materials and Methods section. Both networks 1 and 6 yielded very high z-scores (44.06 and 23.76 respectively), indicating highly enriched PPI in each network. This demonstrates that our QCM approach not only identifies a co-expression module that is highly enriched as a functional module, but also is capable of finding physically interacting networks, which confirmed the previous finding that the co-expression module can reveal those genes that form physically interacting modules [19].

**Table 3:** Comparison of Cancer Network 1 with gene signatures from other cancer microarray studies.

Reference	Cancer type (Sample size)	Signature type	% genes overlapping with Cancer Network 1 genes
[72]	Drosophila cell line and siRNA	Mitotic division	26
[23]	Breast cancer (311 patients)	Cell proliferation signature	41
[22]	Breast cancer (3 datasets)	Cell cycle regulation and proliferation	42
[24]	Breast cancer (347 tumors)	Genetic grade signature	55
[26]	Hepatocellular Carcinoma (91 tumors, 60 normal)	Cell cycle regulation and proliferation	58
[27]	Meningiomas (3 datasets, 10,16, 56)	Tumor grade	64
[28]	Glioblastoma (5 datasets)	Mitotic cell cycle	74
[25]	6 cancer types (12 datasets)	Chromosome instability	80
[9]	Multiple cancer types (23 datasets)	Cell proliferation	90
[67]	13 cancer types (13 datasets)	Cell cycle	100

We also isolated a gene network from cancer datasets that has very diverse GO terms but with no apparent theme (cancer Network 4 with 73 genes, Figure 2, Table 2, Table S1). Genes in this orphan network participate in functions including *small molecule biochemistry, lipid metabolism, cell-to-cell communications, connective tissue development*, etc. Interestingly, an almost identical network is also found in the normal tissue datasets (normal Network 3 with 60 genes, Figure 2, Table 2, Table S2). Inside this gene network, eight genes were involved in DNA damage response (*SMG1*, *GTSE1*, *GTF1H3*, *PMS2P1*, *PMS2L2*, *XRCC2*, *DCLRE1C*, and *UACA*) based on GO term enrichment analysis. *PGF* is involved in angiogenesis, epithelial cell growth, and the migration of mesenchymal stem cells [29], [30]. *NEK9*, *HAUS2* are involved in mitotic spindle formation and centrosome integrity [31], [32]. However, a majority of the genes in this network are not closely connected with each other in the protein-protein interaction database from the most updated Ingenuity Knowledge Base at the time of the manuscript preparation. Instead, they either participate in diverse functions, which are not tightly linked to cancer, or have not been extensively studied. Using the gene set enrichment analysis tool TOPPGene, we found that within this network, 22 were down- regulated in poorly differentiated thyroid carcinoma, 13 were down-regulated in nasopharyngeal cancer, breast cancer and hepatocellular carcinoma (HCC), and 12 were up-regulated in the intrahepatic metastatic HCC versus primary HCC. However, it is not clear how these genes are functionally or physically interacting with each other, and the majority of them have not been linked with cancer development. These genes, along with other less studied members in this network, may be good targets for future cancer studies.

### Networks as a potential prognosis marker for multiple cancers

**Figure 4:**
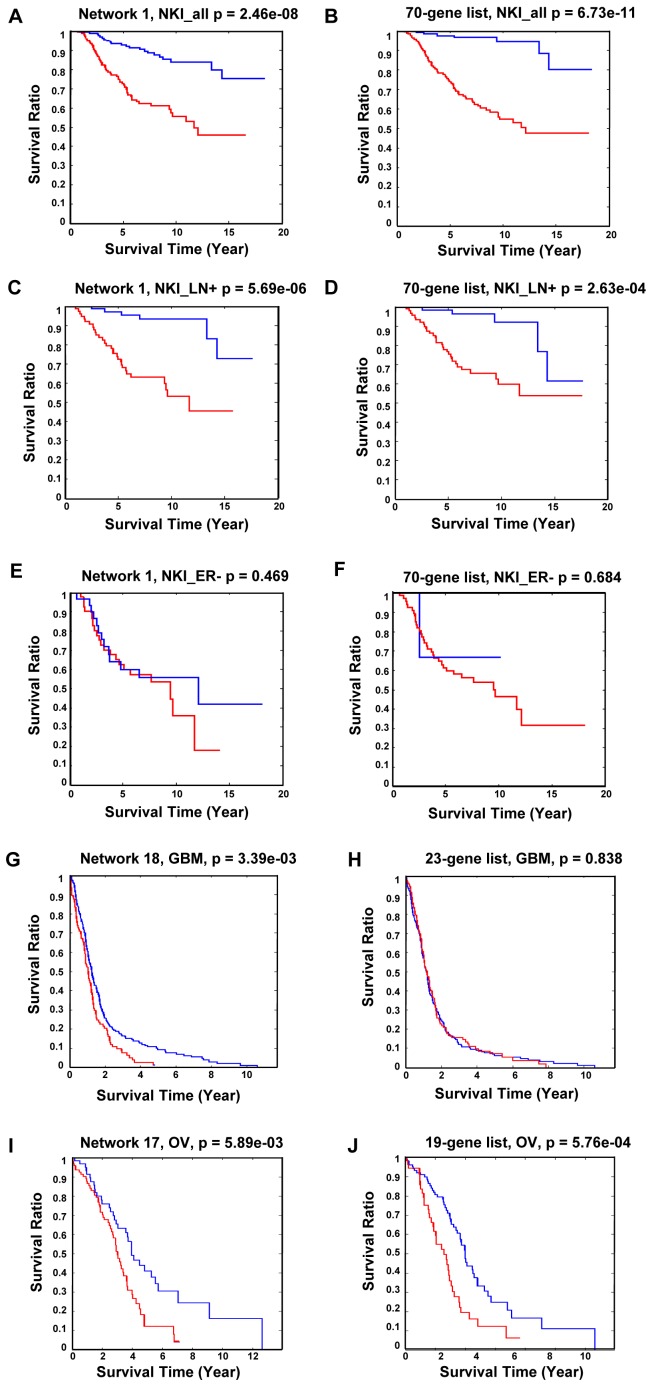
Kaplan-Meier curve of breast cancer, glioblastoma (GBM) and ovarian cancer (OV) using network genes identified from cancer datasets. The p-values are computed using Log- rank test with 100 repeats. A: using Network 1 genes on NKI mixed cohort; B: using Van't Veer 70-gene signature [1] on NKI mixed cohort; C: using Network 1 genes on NKI LN+ cohort; D: using van't Veer 70-gene signature [1] on NKI LN+ cohort; E: using Network1 genes on NKI ER− cohort; F: using Van't Veer 70-gene signature on NKI ER− data. G: using Network 18 genes on TCGA GBM dataset; H: using 23-gene signature on TCGA GBM cohort [28]. I: using Network 17 genes on TCGA OV cohort. J: using 19-gene signature on TCGA OV dataset [34]. Blue lines: good survival outcome group; Red lines: poor survival outcome group. LN+: lymph node positive. ER−: estrogen receptor negative.

Since many gene signatures and biomarkers involved in cell cycle control and cell proliferation overlapped with genes in Network 1 from cancer datasets (Table 3), we tested their prognostic capability in breast cancer, ovarian cancer (OV) and glioblastoma (GBM) patients (Figure 4). Datasets were separated according to Network 1 (Figure 4A, C, E) or according to the Van't Veer 70-gene list (Figure 4B, D, F), and the survival of patients from each set were plotted up to 20 years. For patients from the NKI breast cancer dataset with mixed subtypes as well as the lymph node-positive (LN+) cohort, Network 1 separated the good and poor outcome groups comparably well as the Van't Veer 70-gene signature [1] (Figure 4A–D), and both passed the p-value significance threshold after Bonferroni correction, despite the fact that the two lists only shared five genes in common (*CENPA, MCM6, ORC6L, PRC1, RFC4*). However, for the ER-negative cohort, neither Network 1 genes (Figure 4E) nor Van't Veer 70-genes (Figure 4F) identified the individuals with longer survival. This suggests that the cell-proliferation network is less prognostic for the ER-negative cohort.

For GBM and OV cancer patients, in which prognosis studies based on microarray analysis are relatively scarce, we also tested the networks we identified from multiple cancer datasets. Network 1 genes failed to separate the good and bad outcome groups, even though certain cell proliferation genes are known to be associated with these cancers, such as *ASPM* in GBM [7] and *BRCA1* and *BRCA2* in OV [33]. Thus, a more sophisticated supervised feature selection approach is needed to improve the separation by selecting most relevant genes from this network [34]. However, Network 18 genes, which are enriched with cellular respiration function, had good prognosis power for GBM (p = 5.89E-3, Figure 4G) on the TCGA GBM dataset, while a recently published GBM 23-gene signature [28] failed to separate the good versus poor patient outcome using the same unsupervised K-means clustering approach on this dataset (Figure 4H). For OV patients, Network 17 genes, which have no significantly enriched GO-term (Table 2, Table S1), performed best among all the networks to separate the good and bad outcome groups (p = 3.39E-3, Figure 4I), comparable to an OV 19-gene signature when applied to the same OV dataset (Figure 4J) [34].

### Validation of the predicted gene functions in Network 1 using RNAi

Genome instability, such as aneuploidy, due to hyperactive centrosome duplication (also called centrosome amplification) has been observed for decades in cancer cells [35], [36]. DNA repair proteins have recently been shown to localize and regulate the process as well [37]–[41]. Based on these findings, we then looked in Network 1 for genes with unknown functions to further study their roles in genome stability maintenance. Such genes/proteins have limited numbers of publications, or have not previously been shown to regulate centrosome duplication or homologous recombination. In addition, most genes we selected are absent from the validated PPI network in Figure 3A (red circles indicate the four genes present in the validated PPI network). By silencing the expression of target genes by transfection of siRNA, we screened for cells defective in homology-directed DNA repair (HR) or cells with supernumerary centrosomes. *BRCA1* was used as a positive control, since its functions in homologous recombination and centrosome amplification have been known [37], [40], [42]–[45].

**Figure 5:**
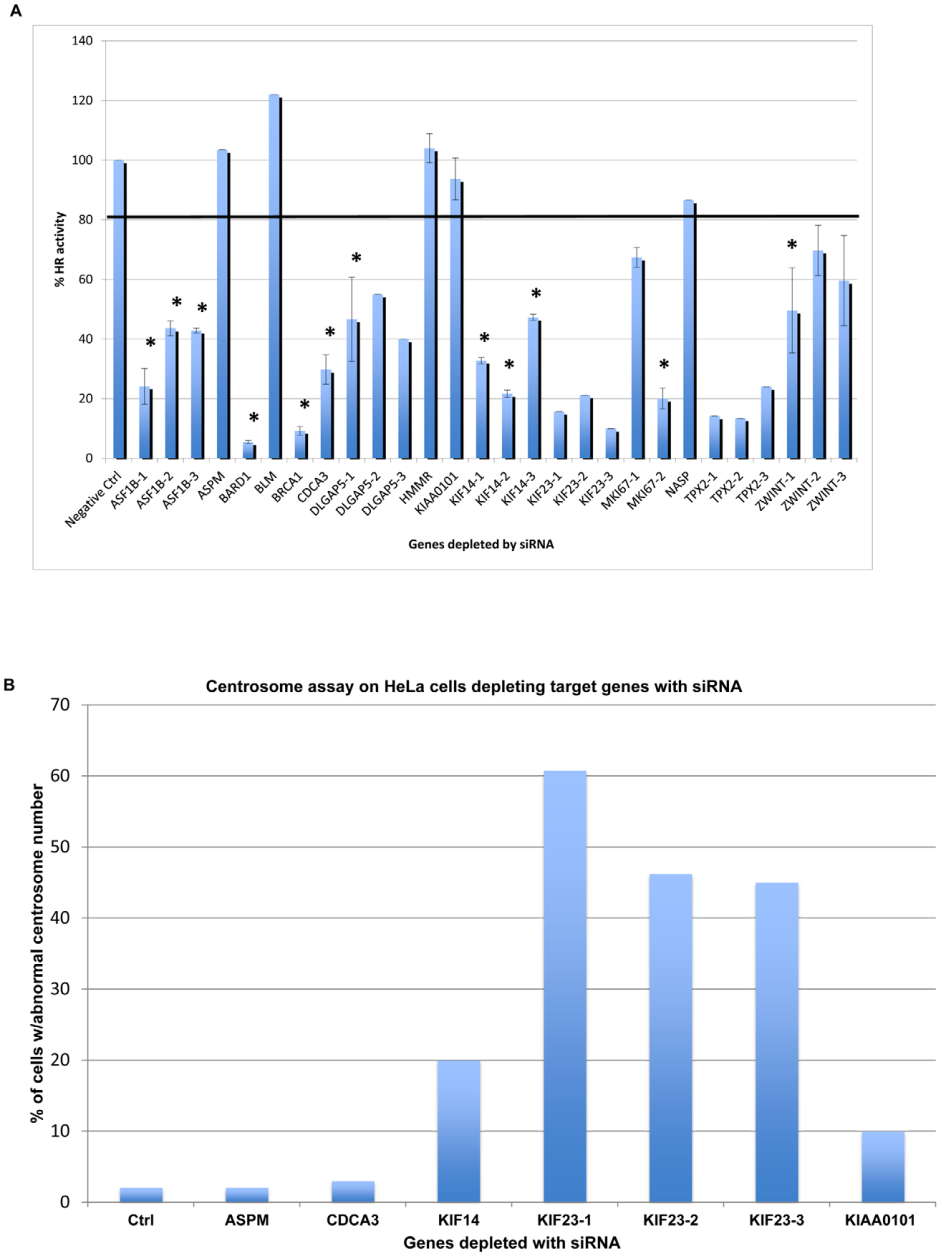
Cell-based assays to test gene involvement in the genome stability maintenance using RNAi. Cells transfected with firefly gene GL2 siRNA were used as the negative control for both assays. A: HR assay on HeLa cells depleting target gene expression by siRNA. Error bar represents standard error. Asterisks indicate the results with statistically significant decreased activity upon siRNA depletion using Student's test (p<0.05). Black line represents the 80% of HR activity in the control sample as a cutoff. B: Centrosome assay on HeLa cell line depleting target gene expression by siRNA. Error bar represents standard error.

Out of the 13 genes we depleted with siRNA besides *BRCA1*, seven were significantly impaired for HR function (*ASF1B*, *BARD1*, *CDCA3*, *DLGAP5*, *KIF14*, *MKI67* and *ZWINT*), and one was marginally impaired for HR function (*NASP*) (Figure 5A, Table 4). Four showed centrosome amplification (*KIAA0101*, *KIF14*, *KIF23* and *HMMR* [5]) on the HeLa cell line and the breast cancer cell line Hs578T (Figure 5B, Table 4, Figure S2). Among these genes, *BARD1* interacts with *BRCA1* in the HR pathway [46], therefore the HR decrease upon BARD1 depletion was expected. BLM is an important genome stability maintenance protein with biochemical activity of a helicase, and BLM suppresses HR [47], [48]. The HR suppression activity of BLM explains why its depletion increased the cell activity of HR. HMMR (hyaluronan-mediated motility receptor), although directly interacts with BRCA1 and BRCA2, surprisingly does not affect HR activity in the cell after being depleted. However, HMMR depleted cells are known to exhibit centrosome amplification phenotype [5]. The depletion of KIAA0101 did not affect the HR activity, but centrosome amplification was observed. The unaffected HR activity upon KIAA0101 depletion was confirmed by a separate work published recently [49]. In that work, KIAA0101 was hypothesized to restrict HR activity. In our further study, KIAA0101 was shown to be over-expressed in breast cancer cells, and interacting directly with the BRCA1 protein [11]. This finding provided strong evidence that the cancer frequent co-expression network mining can be a powerful tool to direct gene function research, especially to facilitate the search for oncogenes and genes closely related to cancer cell activities.

**Table 4:** Summary of effects on genome stability for genes depleted with siRNA using HR and centrosome assay on HeLa cells.

Gene Symbol	Decrease on HDR	Centrosome Amplification
BRCA1 (positive control)	√	√
ASF1B	√	NT
ASPM	×	×
BARD1	√	NT
BLM	×	NT
CDCA3	√	×
DLGAP5	√	NT
HMMR	×	[5]
KIF14	√	√
KIF23	?	√
MKI67	√	NT
NASP	√	NT
TPX2	lethal	lethal
ZWINT	√	NT

√: Decreased HR activity (less than 80% of HR activity of negative control sample) or supernumerary centrosome phenotype was observed in the cell with target gene depletion. ×: No effect observed on cells with target gene depletion. ND: not determined. ?: Decreased HR activity may be due to plasmid transfection inefficiency. Lethal: the depletion is lethal to cells.

The involvements of *ASF1B*, *DLGAP5* and *ZWINT* in HR of the human cell are novel findings. ASF1B is a histone chaperone that facilitates histone deposition and histone exchange and removal during nucleosome assembly and disassembly [50], [51], [52], [53], [54]. *DLGAP5*, also called *DLG7*, is a potential cell cycle regulator that may play a role in carcinogenesis [55], [56], and it was identified in a gene co-expression analysis of multiple cancer datasets previously [9]. ZWINT is part of the MIS12 complex, which is required for kinetochore formation and spindle checkpoint activity [57], [58], and from these functions ZWINT would not be anticipated to function in HR. All four genes have never previously been shown to participate in DNA repair. The new discovery of those genes participating both in spindle/microtubule regulation and HR may explain the high frequency of hits of these genes in multiple gene expression profiling studies of cancer datasets (Table 3). We also tested HR upon TPX2 depletion, and decreased HR activity was observed. However, TPX2 depletion is lethal to cells, therefore it is difficult to determine whether the decrease of HR activity is due to the potential TPX2 function in DNA repair or due to cell death.

MKI67 (also called Ki67) has long been identified as a proliferation marker in breast tumor grading systems. However, the exact function of this protein remains obscure [59]. We found that depletion of MKI67 resulted in up to a five-fold reduction in HR (Figure 5A). This is the first demonstration that MKI67 is required for double-strand DNA break repair. This finding may provide direction for future study of MKI67 to elucidate its role in tumor proliferation.

KIF14 plays an important role in cytokinesis [60]. KIF23 is a plus-end-directed motor enzyme that moves anti-parallel microtubules in vitro. It localizes to the interzone of mitotic spindles. KIF14 and KIF23 directly interact with PRC1 within a complex that also contains KIF4A and KIF20A [60], [61]. KIF14 has been identified as a prognostic marker in breast and ovarian cancer in gene expression profiling studies [9], [62]. KIF23 was also up-regulated with three other genes in non-small cell lung cancer [63]. In our study, KIF14 and KIF23 depleted HeLa cells showed impaired HR, and increased centrosome amplification. However, we found the effect of KIF23 depletion on HR was complicated because its depletion caused cells to become resistant to plasmid transfection, which was confirmed through independent experiments (data not shown). As a result, the KIF23 depletion-induced genome instability is probably due to an indirect effect.

ASPM was hypothesized to regulate spindle formation and mitotic process based on sequence similarity (UniProt), but in our assay, ASPM depleted cells did not have the centrosome amplification phenotype. This indicates the ASPM's role in spindle regulation may be indirect or it participates in different pathways than the above ones.

## Discussion

It is clear that networks identified from cancers and normal tissues are very different. The former contain more tightly connected networks with more members, and with GO terms closely related to cancer-specific biological processes. By contrast, analysis from normal cells reveals fewer gene networks with fewer members that mostly comprise normal cell housekeeping functions. As described in [64], different cancers share common “hallmarks” such as replicative immortality, angiogenesis, invasion and metastasis. Then in [65], four additional properties were proposed as common hallmarks or characteristics for cancers including genome instability/mutation, tumor promoting inflammation, avoiding immune destruction and deregulating cellular energetics. In addition, tumor microenvironment also plays a pivotal role in cancer development. Interestingly, our findings are highly consistent with these common cancer properties. The predominant network identified from multiple cancer datasets is most enriched in genes involved in cell cycle control, genome instability and DNA repair functions (Network 1 with 412 genes), suggesting that regardless of the cancer types, the most active process in the cancer cell is cell proliferation, and genome instability is the enabling characteristics of cancer. Besides the cell cycle control and genome instability networks identified from cancer datasets, several immune/inflammation response networks and the type I interferon network were also identified which are potentially related to the tumor promoting inflammation and avoiding immune disruption characteristics. In addition, the tightly connected extracellular matrix network (Network 6 from cancer, Table 2) identified in cancer datasets supports the importance of tumor microenvironment in cancer development. Lastly, the lack of the cell metabolism network in cancer compared to the normal tissues (Network 1 from normal tissues, Table 2) implies disruption of normal cellular energetic processes. Overall, our results reveal that the common cancer hallmarks and characteristics involve highly coordinated transcriptomic activities. Many of the cancer network genes are differentially expressed in cancer vs. normal samples, and were identified using a differential expression analysis approach. In fact, cancer network 1 includes a high proportion of the cell proliferation genes identified from a differential expression study [22], [23] (Table 3). Some studies combined differential expression analysis with condition specific co-expression network mining [66]–[68], and identified cell cycle/cell proliferation networks in the cancer microarray datasets. Specifically in a smaller scale multiple cancer/normal microarray dataset study using differential co- expression approach, similar but smaller cell cycle networks were identified that were 100% included in our Cancer Network 1 gene list [67] (Table 3). However, the advantage of using a frequent co-expression network mining approach is that it combines datasets from multiple diseases instead of comparing two conditions and therefore, many microarray studies with few or no normal samples can still be integrated in our mining approach even though they are not suitable for differential expression analysis. Furthermore, the network genes identified from frequent co-expression analysis clearly groups genes into functionally and even physically interacting clusters, while differential expression analysis identifies isolated genes which need to be further clustered for functional analysis.

In normal tissues, the two biggest gene networks identified are involved in cell metabolism and protein synthesis; the members are mostly housekeeping genes (Table S2). Because our frequent co-expression network mining algorithm QCM uses gene-pair frequency as the edge weight, tissue-specific genes and networks do not get enriched in this network mining approach. The difference between cancer and normal tissue networks indicates that despite the different tissue sources and different cell types, cancer cells are more similar in their physiological activities, whereas normal cells are more distinct and specific to their own cell- type specific activities.

It has been found that several immune response gene co-expression networks are present in the multiple cancer microarray datasets [9], and this is confirmed in our results, in which the second largest network (Network 2 of 260 genes) is mostly involved in immune response. Protein synthesis is also an important part of cell proliferation, thus the third largest network found in cancer datasets is involved in protein synthesis. In addition, the cancer tissue microenvironment plays a key role in tumorigenesis, tumor development and metastasis. Our search also identified a network of 57 genes (Network 6) that are mostly collagen- related genes, which form an important part of the extracellular matrix and the cancer tissue microenvironment.

The cancer specific networks we identified showed strong prognostic power in breast cancer, glioblastoma, and ovarian cancer patients, especially the cell cycle/proliferation network (Network 1). It outperforms the 70-gene signature in the survival analysis of lymph-node positive cohort, and for a subset of this network (Network 1 before merging step), the performance is even better (Figure S1). However, it is likely that the large size of this network caused problems in the K-means algorithm, and hence the performance was impaired in the GBM and OV prognosis. Instead, smaller networks (Networks 17 and 18), each with only ten gene members, can be useful in GBM and OV prognosis.

It has been shown that chromosomal instability and aneuploidy are typical features of solid tumor cells (reviewed in [69], [70]). Mitotic genes from *Drosophila* have been used to predict survival for breast cancer patients [71]. Genes from Network 1 of cancer datasets are highly enriched for genome stability maintenance functions such as cell cycle, mitotic apparatus assembly and regulation as well as DDR and cell proliferation. The importance of this co- expression network in cancer has been confirmed by its significant overlap with a number of gene signatures for cell proliferation [9], [22], [23], mitotic division and chromosomal instability [25], [72] (Table 3). Among them, the key spindle formation regulator Aurora-A and TPX2 co- expression were observed in increased abundance in several cancer types (reviewed in [73]). This led us to examine genes in that cluster that have not been shown to be directly involved in DDR or genome stability maintenance in human cells. Genes that are verified to play roles in these functions are potential oncogenes. They may serve not only as candidates of biomarkers, but also as molecular targets of anti-cancer drugs, for example, Aurora-A inhibitors are already under clinical trials [74].

The QCM parameter β and γ initial settings affect the number of networks found and the size of networks. As described in the Materials and Methods, γ is the parameter controlling the selection of the first edge in each network, λ and *t* control the adaptive threshold of network density. Together these three parameters guarantee a lower bound of density for all networks. β is the threshold for merging networks. High γ generates fewer networks, and high β generates small and tight (high cluster density) networks. In order to obtain tightly clustered networks with relatively small size, we selected γ = 0.8 and β = 0.8 for cancer datasets, and γ = 0.7, β = 0.8 for normal tissue datasets (to accommodate the smaller sample size in each dataset and less total number of datasets available for normal tissues). However, when β and γ are set to 0.5 or above, the results are highly reproducible, which means the predominant networks we found from cancer datasets are always enriched with the same GO term, i.e., *cell-cycle/cell proliferation network, immune response and protein synthesis*, whereas the networks obtained from normal tissue datasets are always enriched with housekeeping functions such as *cellular respiration* and *protein synthesis*. The small set of core genes are identified with β and γ set to high values, as the values of the parameters decrease, more and more genes join the network, but the core genes and the enriched function are still preserved (Table S3, Figure S3). This suggests that the QCM algorithm is very robust in mining the frequent co-expression network in cancer microarray data. Furthermore, for all the γ settings above 0.5, we found very little overlap for the top three co-expression networks identified between cancer microarray datasets and the ones from normal tissue (see Table S3), which strongly suggests that the gene co-expression clusters found in cancer datasets are specifically involved in cancer-related functions and pathways, while the ones found in normal tissues are not.

As we have demonstrated, the QCM network mining approach can be applied to either single or multiple microarray datasets for co-expressed gene clusters. However, there are some intrinsic limitations not only for this QCM algorithm, but also for the co-expression network mining in general. In order to obtain a high level of significance for the Pearson correlation computing between each pair of genes, the dataset has to be in a relatively large size, and contain a good proportion of genes with significant signals readings and variations. Also due to the focus on gene expression correlation study, or transcriptome profiling study, any interaction in the non-transcriptional level, such as interactions in the post-transcription, translation and post-translation as well as DNA replication, will not be captured. This is the major limitation of the co-expression network mining approach *per se*. Another drawback exists in our current workflow is that we chose Pearson correlation to measure the correlation between any gene pair, which is fast in the computing step. However, in a biology system, the relationship between the expressions of two genes can be non-linear as well, therefore we plan to test an improvement to the method by incorporating the Spearman rank correlation and mutual information (MI) to further investigate and extract the non-linear correlated co- expression clusters among genes.

## Materials and Methods

### Cancer and normal tissue microarray dataset selection

The NCBI Gene Expression Omnibus (GEO) was queried for cancer microarray datasets prepared from various types of primary tumor biopsy samples, with a sample size of 30 or more in a specific dataset (Table 1). This resulted in 27 cancer microarray datasets of 33 cancer types, including sarcoma, carcinoma, adenocarcinoma, leukemia, lymphoma, as well as brain cancer. For datasets containing normal tissue control samples, they were removed prior to further co-expression network mining. At the same time, we also queried the GEO database for various types of normal tissue microarray with sample sizes of at least 20 for any tissue type, resulting in 7 datasets composed of 9 types of normal tissues (Table 1). If a normal tissue dataset contained diseased tissue data, they were removed before running network mining. For datasets containing multiple tissue types, they were separated into different datasets before computing PCC. The cancer and normal tissue datasets were all from the Affymetrix GPL570 platform to avoid any platform related systematic errors among the datasets. The tissue and cancer types were carefully chosen to avoid bias towards a particular type of cancer or tissue. All datasets were pre-filtered to remove probes without gene annotation, and for genes with multiple probes, we selected the one with the highest expression values. This resulted in 20,827 probes/genes.

### Frequent gene co-expression network mining using QCM

Each pair of genes from a specific cancer or normal tissue microarray dataset were computed for Pearson Correlation Coefficient (PCC), and only the gene pairs with high |PCC| values were retained for network construction. However, since the range of |PCC| values varies substantially among different datasets, we cannot select a uniform threshold on the |PCC| values. Instead, we adaptively set the threshold for |PCC| values to the top 5% (95 percentile) in each dataset to select the ones with high confidence (all the selected PCC have p-values less than 0.05). The frequency of such gene pairs in either cancer datasets or normal datasets was used as the edge weight for network mining using a greedy quasi-clique discovery algorithm called Quasi-Clique Merger (QCM) [16]. QCM is an iterative greedy algorithm. At the initial step, the edge with largest weight in the entire work is identified and its weight is designated as 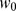. Then for every iterative step, a new network is established with the first edge being the edge with the largest weight that is not contained in any previously established networks. In addition, the weight of this network cannot be smaller than 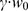 (0<γ<1), otherwise the program stops. Once the first edge for a network is identified, new edges which can contribute most to the total density of the selected network will be added one a time. During this process, the density of selected network will gradually reduce. The process will stop if the edge of choice will drive the density of the network below an adaptive threshold defined by two parameters *t* and λ. Once the iteration is over, networks with overlap ratio above a re-defined threshold β will be merged iteratively and form a large network. The overlap ratio is defined as the ratio between the number of shared genes between two networks and the number of genes in the smaller network. The algorithm was implemented in C++, with the hierarchical clustering step omitted. The parameters were set as follows: *t* = 1.0, β = 0.8–0.9, λ = 2.0, γ = 0.5–0.9. The density of a weighted network with *N* vertices was defined as: 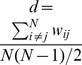 with *w* *_ij_* being the weight between vertices *v* *_i_* and *v* *_j_* (*i = 1, 2, …, N*; *j = 1, 2, …, N*; *i* *≠j*), normalized between 0 and 1. For randomly selected gene subsets, average gene subset size 10 and 400 were selected from the entire gene pool of Affymetrix HU133 2.0 Plus platform (GEO accession number GPL570), and the network density for each subset was computed using above formula. The random selections were repeated 1000 times for each size, and the average network density was calculated.

### Homology directed repair (HR) assay

HeLa-DR13-9 cells (Puro^R^) and the pCBASce vector (Amp^R^) containing disrupted GFP gene and I-SceI were used in the assay as described in [42], [75]. Cells transfected with firefly gene GL2 siRNA were used as the negative control. For the experiment with target gene depletion by RNAi, 1 to 3 independent siRNA molecules were used for each gene (Table S6). The assay was repeated at least three times for each siRNA depletion. Two rounds of transfection were performed following Oligofectamine+siRNA protocol (Invitrogen). On Day 1, HeLa-DR cells (4×10^4^ in a 2 cm^2^ well) were plated in media of DMEM with 1% Pen/Strep, 10% Bovine Serum and Puromycin final conc. of 1.5 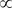
g/ml. On Day 2, the first transfection was performed with 60 pmoles of siRNA with 1.5 µL of Oligofectamine. On Day 3, the cells were transferred to 10 cm^2^ well dishes. On Day 4, 100 pmoles of siRNA with 3 µg of pCBASCeI expression vector were co- transfected. On Days 5 to 7, the cells were trypsinized and those among 10,000 total cells that expressed green fluorescence were measured using a Becton Dickinson FACSCalibur instrument in the Ohio State Comprehensive Cancer Center's Analytical Flow Cytometry core lab. The pCAGGS vector was used as a control. Both pCBASce and pCAGGS were gifted from M. Jasin of the Memorial Sloan-Kettering Cancer Center.

### Centrosome duplication assay

The assay was done according to [11] on HeLa and Hs578T cell lines. 1 to 3 independent siRNA molecules were used for each gene (Table S6). siRNA and GFP-centrin plasmid [76] transfection was done using Lipofectamine 2000 (Invitrogen) according to the manufacturer's protocol, and cells were fixed 48 hours post- transfection. Either one or three different siRNA were transfected for a target gene. GFP- centrin2 marks centrioles, and these were counted by fluorescence microscopy using a Zeiss Axiovert 200 M microscope. The same GL2 siRNA transfected cells were used as the negative control.

### Survival analysis

The Breast Cancer dataset (NKI-295 dataset) and clinical information were obtained from the Netherlands Kanker Instituut (NKI) with 295 patients (226 ER+ and 69 ER−, 147 LN− and 148 LN+). The Glioblastoma multiforme (GBM) and ovarian serous cystadenocarcinoma (OV) dataset was downloaded from the TCGA website (http://tcga.cancer.gov/). Among them, 345 patients from GBM and 156 from OV with valid vital status information were used.

For a selected gene list, the gene expressions of a patient form a vector. For testing datasets from different microarray platform, only matched genes from identified networks were used. We then used a K-means clustering algorithm (with distance set as correlation, repeated 100 times) to cluster patients into two groups. The survival time statistics were calculated by log rank and visualized in Kaplan-Meier survival curves [77]. If a patient's vital status is ‘LIVING’, ‘days_to _last_followup’ was used for the survival curve, otherwise, the ‘days_to_death’ was used.

### Gene ontology enrichment analysis, protein-protein interaction network construction and PPI enrichment analysis

GO enrichment on each the networks identified from QCM was analyzed by ToppGene Suite developed by the Division of Biomedical Informatics, Cincinnati Children's Hospital Medical Center (*BMI CCHMC*) (URL http://toppgene.cchmc.org). PPI networks were constructed using Ingenuity® Systems (IPA, http://www.ingenuity.com) with only validated physical protein-protein interactions extracted from the Ingenuity Knowledge Base using cancer network genes as input. PINA (**P**rotein **I**nteraction **N**etwork **A**nalysis) data were used to compute the significance of protein-protein interactions in a specific network gene set. PINA integrates protein-protein interaction data from six curated public PPI databases and builds a comprehensive, non-redundant protein interaction dataset to look for interacting gene pairs [78]. For a cancer network being tested, we first query its genes in PINA database for known PPI relationship, and the significance of the number of hits in the PINA database was measured using hypogeometric test implemented in Matlab. Total of 73,472 gene pairs from PINA was used in the hypogeometric test. In addition, we also compared the tested cancer network with randomly selected networks. Specifically, we generated a randomly selected gene list (from the entire gene set of Affymetrix GPL570 platform) with the same number of genes as the cancer network, and then queried in PINA database for this random list and counted how many hits (known PPI relationships in PINA) can be detected. This random test was then repeated 500 times and the number of hits in the 500 tests was used to estimate a null distribution of PPI hits in PINA database. It was then used to compute the z-score for the number of hits for the two true cancer networks (network 1 and network 6). The z-score is the measurement of how many standard deviations the observed value is away from the mean, indicating the statistical significance of PPI enrichment in this case.

## Supporting Information

Figure S1 Kaplan-Meier curve on NKI breast cancer datasets using the core network 1 genes before merging step.
(PDF)

Figure S2 Centrosome assay on breast cancer cell line Hs578T depleting target genes using siRNA.
(PDF)

Figure S3 Overlaps among cancer network 1 identified from different QCM parameter settings.
(PDF)

Table S1 Merged networks identified from cancer primary tumor microarray datasets (β = 0.8, γ = 0.8, λ = 2.0, t = 1.0).
(PDF)

Table S2 Merged networks identified from normal tissue microarray datasets (β = 0.8, γ = 0.7, λ = 2.0, t = 1.0).
(PDF)

Table S3 Comparison of the top three major networks identified from cancer vs. normal tissue microarray datasets using different parameter settings.
(PDF)

Table S4 Details of the networks identified from lung cancer microarray datasets using different parameter settings.
(PDF)

Table S5 Details of the networks identified from normal lung tissue microarray datasets using different parameter settings.
(PDF)

Table S6 The sequences of siRNA probes used for target gene depletion in HeLa and Hs578T cell lines.
(PDF)
