# Supplementary material for: Weighted Frequent Gene Co-expression Network Mining to Identify Genes Involved in Genome Stability
Source: PLoS Comput Biol. 2012 Aug 30;8(8):e1002656. doi: 10.1371/journal.pcbi.1002656 (PMC3431293; doi:10.1371/journal.pcbi.1002656)

Figure S1: Kaplan-Meier curve on NKI breast cancer datasets using the core network 1 before merging step. Blue: good survival outcome group; red: poor survival outcome group.

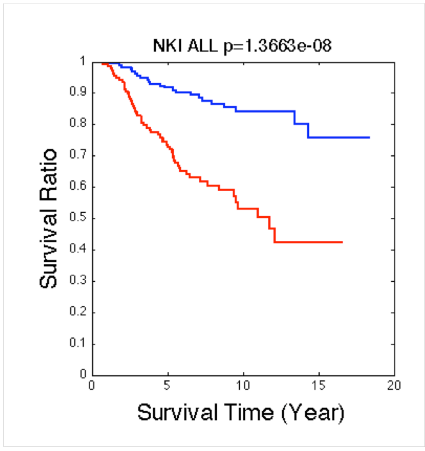

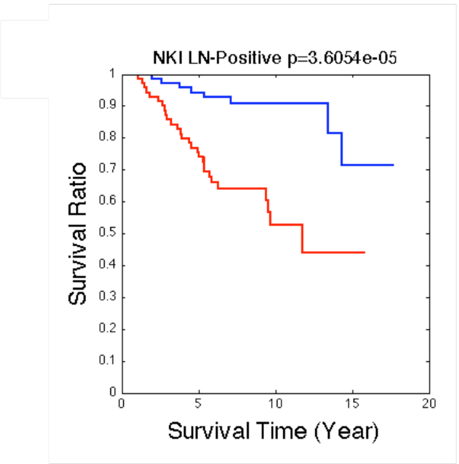

Supplement: Figure S1 — Kaplan-Meier curve on NKI breast cancer datasets using the core network 1 genes before merging step. (PDF) [file pcbi.1002656.s001.pdf]
