# Supplementary figures and images for: Weighted Frequent Gene Co-expression Network Mining to Identify Genes Involved in Genome Stability
Source: PLoS Comput Biol. 2012 Aug 30;8(8):e1002656. doi: 10.1371/journal.pcbi.1002656 (PMC3431293; doi:10.1371/journal.pcbi.1002656)

**Figure S2: Centrosome assay on breast cancer cell line Hs578T depleted of target genes using siRNA.**

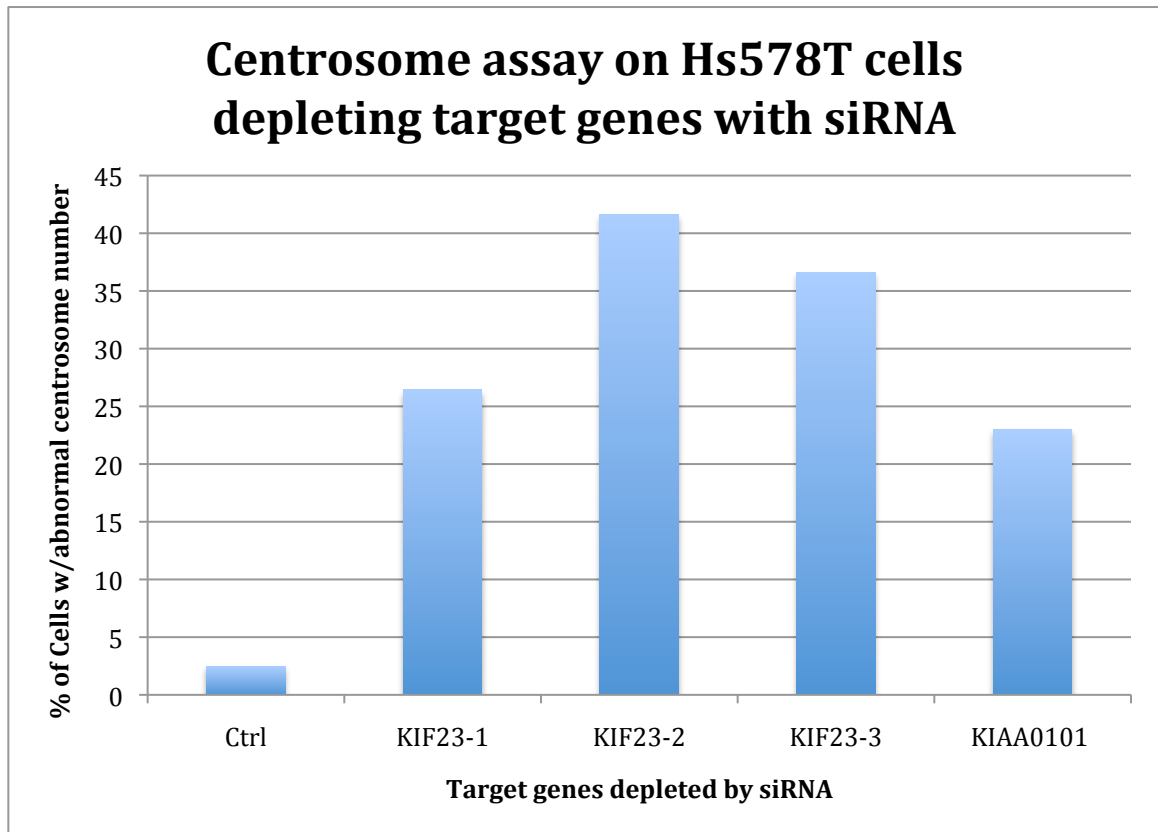

Supplement: Figure S2 — Centrosome assay on breast cancer cell line Hs578T depleting target genes using siRNA. (PDF) [file pcbi.1002656.s002.pdf]
