# Supplementary material for: Weighted Frequent Gene Co-expression Network Mining to Identify Genes Involved in Genome Stability
Source: PLoS Comput Biol. 2012 Aug 30;8(8):e1002656. doi: 10.1371/journal.pcbi.1002656 (PMC3431293; doi:10.1371/journal.pcbi.1002656)

Figure S3: Overlaps among cancer network 1 identified from different QCM parameter settings. The numbers in each the upper row indicate the size of the network after merging. The two numbers in the lower row are γ and β values respectively for each QCM mining results. The overlapping area of any two circles indicates the number of common genes shared with the two networks.

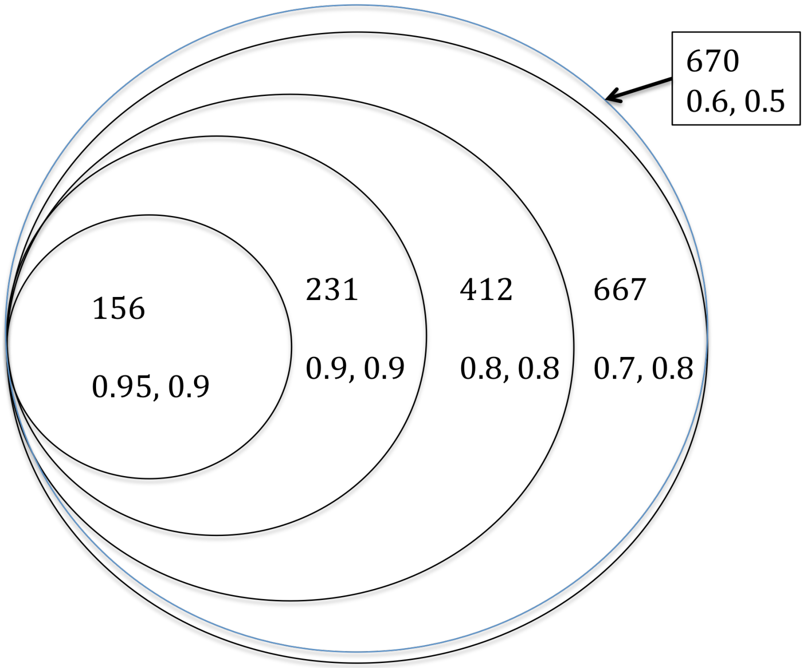

Supplement: Figure S3 — Overlaps among cancer network 1 identified from different QCM parameter settings. (PDF) [file pcbi.1002656.s003.pdf]
