# Supplementary material for: Weighted Frequent Gene Co-expression Network Mining to Identify Genes Involved in Genome Stability
Source: PLoS Comput Biol. 2012 Aug 30;8(8):e1002656. doi: 10.1371/journal.pcbi.1002656 (PMC3431293; doi:10.1371/journal.pcbi.1002656)
Supplement: Table S1 — Merged networks identified from cancer primary tumor microarray datasets (β = 0.8, γ = 0.8, λ = 2.0, t = 1.0). (PDF) [file pcbi.1002656.s004.pdf]

Table S1: Merged networks identified from cancer primary tumor microarray datasets (β=0.8, =0.8, λ=2.0, t=1.0)

Network ID	Network size in merged network	Top biological processes in the merged network	GO-term enrichement p-value	Gene member
1	412	Cell cycle	6.30E-130	ACTL6A, ADSL, ANLN, AOF2, ARHGAP11A, ARL6IP6, ASF1B, ASPM, ATAD2, ATIC, AURKA, AURKB, BARD1, BIRC5, BLM, BRCA1, BUB1, BUB1B, BXDC2, C11orf82, C12orf48, C13orf3, C13orf34, C15orf23, C16orf59, C16orf75, C18orf24, C1orf112, C21orf45, C2orf47, C3orf17, C3orf26, C4orf46, C5orf34, C6orf173, C9orf40, CACYBP, CASC5, CASP2, CCDC34, CCDC5, CCDC99, CCNA2, CCNB1, CCNB2, CCNE2, CCNF, CCT3, CCT4, CCT5, CCT6A, CCT7, CDC2, CDC20, CDC25A, CDC25C, CDC45L, CDC6, CDC7, CDCA2, CDCA3, CDCA4, CDCA5, CDCA7, CDCA8, CDK2, CDKN3, CDT1, CENPA, CENPE, CENPF, CENPH, CENPI, CENPK, CENPL, CENPM, CENPN, CENPO, CENPQ, CEP152, CEP55, CEP78, CHAC2, CHAF1A, CHAF1B, CHEK1, CIP29, CKAP2, CKAP2L, CKAP5, CKS1B, CKS2, CNOT10, COMMD2, CPSF3, CSE1L, CTPS, DBF4, DDX39, DDX46, DEK, DEPDC1, DEPDC1B, DGUOK, DHFR, DIAPH3, DKC1, DLGAP5, DNA2, DNAJC9, DNMT1, DONSON, DSCC1, DSN1, DTL, DTYMK /// LOC727761, E2F1, E2F2, E2F8, EBNA1BP2, ECT2, EIF2S2, ELAVL1, ERCC6L, ESPL1, EXO1, EXOSC8, EXOSC9, EZH2, FAM136A, FAM54A, FAM64A, FAM72A /// FAM72B /// GCUD2, FAM83D, FANCD2, FANCG, FANCI, FARSB, FASTKD2, FBL, FBXO5, FEN1, FIGNL1, FOXM1, GEMIN6, GGH, GINS1, GINS2, GMNN, GMPS, GNL2, GTF3C3, H2AFX, H2AFZ, HAT1, HELLS, HJURP, HMGB2, HMMR, HNRNPA2B1, HNRNPC, HNRNPR, HPRT1, HSD17B10, HSPB11, HSPD1, HSPE1, ILF2, INTS7, ITGB3BP, KIAA0101, KIAA1524, KIF11, KIF14, KIF15, KIF18A, KIF18B, KIF20A, KIF20B, KIF22, KIF23, KIF2C, KIF4A, KIFC1, KNTC1, KPNA2 /// LOC728860, LIG1, LMNB1, LMNB2, LOC100134172 /// PRIM2, LOC731049 /// UBE2S, LRPPRC, LSM2, LSM3, LSM4, MAD2L1, MAGOH, MAGOHB, MAPKAPK5, MASTL, MCM10, MCM2, MCM3, MCM4, MCM5, MCM6, MCM7, MCM8, MELK, MKI67, MKI67IP, MLF1IP, MND1, MPHOSPH9, MRPL11, MRPL3, MRPL30, MRPL36, MRPL37, MRPL42, MRPL47, MRPS17, MRPS22, MRPS30, MSH2, MSH6, MTCH2, MTHFD1, MTIF2, MTX2, MYBL2, NANP, NASP, NCAPD2, NCAPD3, NCAPG, NCAPG2, NCAPH, NCL, NDC80, NEIL3, NEK2, NIF3L1, NIT2, NME1 /// NME2, NOL10, NOP56, NRM, NUDT1, NUF2, NUP155, NUP160, NUP188, NUP205, NUP35, NUP37, NUP54, NUSAP1, NXT1, OIP5, OLA1, ORC1L, ORC6L, PAICS, PARL, PAXIP1, PBK, PCNA, PDSS1, PES1, PFDN2, PHB, PKMYT1, PLK1, PLK4, POLA1, POLA2, POLD1, POLD3,

				POLE2, POLQ, POLR2D, POLR2F, POLR2H, PPIH, PPIL5, PRC1, PRIM1, PRMT1, PSMA2, PSMA3, PSMA4, PSMA5, PSMC3, PSMG4, PTGES3, PTTG1, PTTG3, RACGAP1, RAD18, RAD51, RAD51AP1, RAD54B, RAD54L, RAN, RANBP1, RBBP8, RBM4, RCC2, RCCD1, RECQL4, RFC2, RFC3, RFC4, RFC5, RG9MTD1, RMI1, RNASEH2A, RPA1, RPP40, RRM1, RRM2, RRP1B, RUVBL1, SAAL1, SAC3D1, SAE1, SERBP1, SFRS1, SFRS10, SFRS2, SFRS7, SFRS9, SGOL2, SHCBP1, SIP1, SKP2, SLBP, SMC1A, SMC2, SMC3, SMC4, SNRPA, SNRPA1, SNRPB, SNRPB2, SNRPC, SNRPD1, SNRPD3, SNRPE, SNRPF, SNRPG, SPAG5, SPC24, SPC25, SR140, SSRP1, STIL, STOML2, SUV39H2, SUZ12, TACC3, TAF5, TARS, TCF19, TCP1, TDP1, TFAM, THOC4, TIMELESS, TIPIN, TK1, TMEM194A, TMEM48, TMPO, TOMM5, TOP2A, TOPBP1, TPRKB, TPX2, TRIP13, TROAP, TSEN15, TTF2, TTK, TYMS, UBA2, UBE2C, UBE2N, UBE2T, UCHL5, UCK2, UHRF1, UMPS, UNG, USP1, USP39, VRK1, WDHD1, WDR12, WDR3, WDR51A, WHSC1, XPO1, XPO5, XRCC5, ZC3H8, ZMYND19, ZNF207, ZNF367, ZWILCH, ZWINT
2	260	Immune response	1.67E-57	ADAP2, ADORA3, AIF1, ALOX5, ALOX5AP, AMICA1, AOAH, APBB1IP, APOBEC3G, ARHGAP15, ARHGAP30, ARHGAP9, ARHGDIB, ARRB2, BCL2A1, BIN2, BTK, C10orf54, C1QA, C1QB, C1QC, C1orf162, C1orf38, C1orf54, C3AR1, C5AR1, CALHM2, CAPG, CARD16, CARD16 /// CASP1, CASP1, CCL8, CCR1, CCR2 /// FLJ78302, CCR5 /// LOC727797, CD14, CD163, CD180, CD2, CD300A, CD300LF, CD33, CD37, CD3D, CD4, CD48, CD52, CD53, CD68 /// EIF4A1, CD72, CD84, CD86, CECR1, CLEC10A, CLEC4A, CLEC7A, CMKLR1, CORO1A, CPVL, CRTAM, CSF1R, CSF2RB, CTSB, CTSD, CTSS, CXCL9, CYBB, CYTH4, DEF6, DOCK2, DOCK8, DOK2, DPEP2, DPYD, ENG, EOMES, EVI2A /// EVI2B, EVI2B, F13A1, FAM26F, FAM78A, FCER1G, FCGR1A /// FCGR1C, FCGR1B, FCGR2A, FCGR2B, FCGR2C, FCGR3A /// FCGR3B, FCGR3B, FERMT3, FGD2, FGD3, FGL2, FGR, FKBP15, FLI1, FMNL1, FOLR2, FPR1, FPR3, FYB, GAB3, GGTA1, GIMAP1, GIMAP2, GIMAP4, GIMAP5, GIMAP6, GIMAP7, GIMAP8, GM2A, GMFG, GMIP, GPR18, GPR183, GPR65, GPR84, GPSM3, GPX1, GRN, GVIN1, GZMA, GZMK, HAVCR2, HCK, HCLS1, HCST, HEXB, HK3, HMHA1, HVCN1, ICAM3, IFI30, IGSF6, IKZF1, IL10RA, IL15, IL18BP, INPP5D, IRF8, ITGAL, ITGAM, ITGAX, ITGB2, KIAA1949, KLHL6, LAIR1, LAPTM5, LAT2, LCP1, LCP2, LGALS9, LGMN, LILRA6 /// LILRB3, LILRB1, LILRB2, LILRB3, LILRB4, LILRB5, LIPA, LOC100130633 /// ZMYM6, LST1, LY86, LY96, LYZ, MAFB, MAN2B1, 1-Mar, MARCO, MFNG, MFSD1, MGAT1, MLKL, MNDA, MPEG1, MRC1 /// MRC1L1, MS4A14, MS4A4A, MS4A6A, MS4A7, MSR1, MYO1F, NAGK, NCF2, NCKAP1L, NLRC3, NPL, OSCAR, P2RY13, PARVG, PIK3AP1, PIK3CD, PILRA, PLA2G7, PLCG2, PLEK, PLEKHO2, PLTP, PLXND1, PPM1M, PSTPIP1, PTAFR, PTPN22, PTPN6, PTPRC, PTPRCAP,

				RAC2, RASSF4, RASSF5, RGS18, RHOG, RIN3, RNASE6, RNF130, RNF166, SAMHD1, SAMSN1, SASH3, SELL, SELPLG, 1-Sep, SH2B3, SIGLEC1, SIGLEC10, SIRPB2, SLA, SLAMF8, SLC15A3, SLC7A7, SLCO2B1, SMAP2, SPI1, SRGN, STAB1, STK10, STX11, TBC1D10C, TBXAS1, TCN2, TFEC, TLR1, TLR2, TLR4, TLR8, TMC8, TMEM140, TMEM149, TNFAIP8, TNFAIP8L2, TNFRSF1B, TNFSF13B, TRAC, TRBC1, TRBC1 /// TRBC2, TREM2, TYROBP, VAV1, VSIG4, WAS, WIPF1
3	136	Protein Synthesis	1.36E-138	AHCY, APEX1, ATP5G2, BTF3, C12orf57, C17orf45, C20orf199, C4orf14, C5orf26, C6orf48, CCDC72, CCNB1IP1, CCNG1, COMMD6, EEF1B2, EEF1G, EEF2, EIF3D, EIF3E, EIF3F, EIF3L, EIF4B, FAU, GAS5, GEMIN7, GLTSCR2, GNB2L1, IGBP1, IMPDH2, LOC100128140 /// LOC390183 /// LOC442162 /// RPS4X, LOC100128936 /// RPL10A, LOC100130553 /// RPS18, LOC100130624, LOC100131713 /// LOC283412 /// LOC284064 /// LOC391019 /// LOC643531 /// LOC647285 /// LOC728820 /// RPL29 /// RPL29P4, LOC100131713 /// RPL29 /// RPL29P4, LOC388474 /// LOC401640 /// LOC441034 /// LOC644029 /// LOC728139 /// LOC728179 /// RPL7A /// RPL7AP11, LOC388524 /// LOC653162 /// RP11-556K13.1 /// RPSA, LOC390354 /// RPL18A, LOC441533, LOC642741, LOC644315 /// RPS7, LOC649299 /// RPL36A, LOC653737 /// LOC729402 /// RPL21, LOC728179, LOC728453, LSM7, MGC87895 /// RPS14, MRPL45, MRPS27, NACA, NAP1L1, NOB1, NONO, NPM1, PFDN5, PHB2, POLR1D, PSMB7, RBMX, RPL10, RPL10A, RPL11, RPL12, RPL13, RPL13A, RPL14, RPL14 /// RPL14L, RPL15, RPL17, RPL18, RPL19, RPL22, RPL23, RPL24, RPL27, RPL28, RPL29 /// RPL29P4, RPL3, RPL30, RPL31, RPL32, RPL35, RPL35A, RPL36, RPL36A, RPL37A, RPL38, RPL4, RPL41, RPL6, RPL7, RPL7A, RPL9, RPLP0, RPLP0 /// RPLP0-like, RPLP1, RPLP2, RPS10, RPS10L, RPS11, RPS12, RPS14, RPS15, RPS15A, RPS16, RPS17, RPS17L4, RPS19, RPS2, RPS20, RPS21, RPS23, RPS24, RPS25, RPS27, RPS28, RPS29, RPS3, RPS3A, RPS4X, RPS5, RPS6, RPS8, RPS9, RSL1D1, SF3B5, SLC25A5, SLC25A6, SNRPD2, SNRPE, ST13, THG1L, TINP1, UBA52, hCG_2015956 /// LOC100127893 /// LOC100130892 /// LOC100131085 /// LOC388401 /// LOC441896 /// LOC641900 /// LOC728380 /// LOC728843 /// RPL7, hCG_21078 /// RPL27A
4	73	No significantly Enriched BF	n.a.	ADAM33, ALMS1, ATF7IP, C18orf45, C7orf28B, C9orf64, CCDC152, CDC2L5, CEP27, CMBL, DBT, DCLRE1C, DDX59, DIP2A, DKFZP434B2016, DLGAP4, FAM161B, FAM63A, FBXW12, FLJ12151, FLJ34077 /// LOC728558, FLJ35848, GTF2H3, GTSE1, HCG2P7, KIAA0894, KIAA1245 /// NBPF1 /// NBPF10 /// NBPF11 /// NBPF14 /// NBPF16 /// NBPF20 /// NBPF3 /// NBPF8 /// NBPF9 /// RP11-94I2.2, LOC100128510, LOC100131096, LOC100132832 /// PMS2L1 /// PMS2L2, LOC152719,LOC23117 /// LOC642799 /// LOC729602, LOC340085, LOC440354 /// LOC595101 /// LOC641298 /// LOC728423 /// LOC729513 /// SMG1, LOC441258,

				LOC493754, LOC51057, LOC641298, LOC645431, LOC647070, LOC654780, LOC728153, LOC728678, LOC728806, LOC90834, LRRFIP1, NEK8, NLN, NUMBL, ORAI2,PDE4C, PECR,PGF, PODNL1, PRR11, RASEF, RG9MTD3, RP5-886K2.1, SH2B2, SLC22A3, SLC35E1,SPN, SUZ12P, TSR1, UACA, UBXN2A, WBSCR23, XRCC2, ZC3H7B, ZNF160,ZNF486, ZNF611, chromosome 1 open reading frame 191
5	61	Type I interferon mediated signaling	8.42E-37	ADAR, BATF2, BST2, C19orf66, CASP1, CMPK2, CXCL10, CXCL11, DDX60, DDX60L, DTX3L, EIF2AK2, EPSTI1, GBP5, HERC5, HERC6, IDO1, IFI27, IFI35, IFI44, IFI44L, IFI6, IFIH1, IFIT1, IFIT2, IFIT3, IRF7, IRF9, ISG15, LAP3, MX1, MX2, NMI, OAS1, OAS2, OAS3, OASL, PARP12, PARP14, PARP9, PLSCR1, PSMB8, PSMB9, PSME2, RSAD2, RTP4, SAMD9, SAMD9L, SP100, SP110, STAT1, STAT2, TAP1, TRIM21, TRIM22, TRIM34 /// TRIM6 /// TRIM6-TRIM34, TRIM69, UBA7, UBE2L6, USP18, XAF1
6	57	Extracellular matrix organization	1.73E-22	ADAMTS12, ANGPTL2, ANTXR1, BGN, CALD1, CD248, CDH11, COL10A1, COL11A1, COL12A1, COL15A1, COL1A1, COL1A2, COL3A1, COL4A1, COL4A2, COL5A1, COL5A2, COL6A1, COL6A2, COL6A3, CRISPLD2, CTHRC1, CTSK, DCN, EDNRA, EMILIN1, ENTPD4 /// LOXL2, FAP, FBLN1, FBN1, FN1, FNDC1, FSTL1, GLT8D2, GPX8, HEG1, HIC1, ISLR, ITGB1, LOXL1, LTBP2, LUM, MARVELD1, MMP2, MRC2, MXRA8, NID1, NID2, PCOLCE, PDGFRB, POSTN, RAB31, RCN3, SPARC, THBS2, TIMP3
7	45	Humoral immune response	1.07E-19	IGH@ /// IGHA1 /// IGHA2 /// IGHD /// IGHG1 /// IGHG2 /// IGHG3 /// IGHM /// IGHV4-31 /// IGHV@ /// LOC100126583 /// LOC100133739, LOC100134331 /// LOC642131 /// LOC652128 /// VSIG6 IGH@ /// IGHA1 /// IGHA2 /// IGHD /// IGHG1 /// IGHG3 /// IGHG4 /// IGHM /// IGHV4-31 /// LOC100126583 /// LOC100134331 /// LOC642131 /// LOC652128 /// VSIG6, LOC652494, IGHV7-81 /// LOC100133739 /// LOC390530 /// LOC642131, IGH@ /// IGHA1 /// IGHA2 /// IGHV3OR16-13 /// LOC100126583, IGH@ /// IGHD /// IGHG1 /// LOC100126583 /// LOC100134331 /// LOC652128, IGH@ /// IGHG1 /// IGHG2 /// IGHM /// IGHV4-31, IGHA1 /// IGHD /// IGHG1 /// IGHG3 /// IGHM /// IGHV4-31 /// IGHV@ /// LOC100126583, IGHA1 /// IGHD /// IGHG1 /// IGHM /// IGHV4-31 /// IGHV@, IGHA1 /// IGHG1 /// IGHG3 /// IGHM /// IGHV4-31 /// IGHV@ /// LOC100133739, IGHA1 /// IGHG1 /// IGHG3 /// IGHM /// LOC652494, IGHA1 /// IGHG1 /// IGHV@, IGHA1 /// IGHG1 /// LOC100133862, IGHM, LOC100133862, LOC652494, IGJ, IGK@ /// IGKC, IGK@ /// IGKC /// IGKV1-5 /// LOC647506 /// LOC652694, IGK@ /// IGKC /// IGKV3-20 /// IGKV3D-11 /// IGKV3D-15 /// LOC440871, IGK@ /// IGKC /// LOC647506 /// LOC650405 /// LOC652493, IGK@ /// IGKV3-20 /// IGKV3D-11 /// IGKV3D-15 /// LOC440871, IGKC, IGKV1-5 /// LOC100130100 /// LOC647506 /// LOC650405 /// LOC652493 /// LOC652694, IGKC /// IGKV1-5 /// LOC647506 /// LOC652694, IGKV4-1, IGL@, IGLC1 ///

7	45	Humoral immune response	1.07E-19	IGH@ /// IGHA1 /// IGHA2 /// IGHD /// IGHG1 /// IGHG2 /// IGHG3 /// IGHM /// IGHV4-31 /// IGHV@ /// LOC100126583 /// LOC100133739, LOC100134331 /// LOC642131 /// LOC652128 /// VSIG6 IGH@ /// IGHA1 /// IGHA2 /// IGHD /// IGHG1 /// IGHG3 /// IGHG4 /// IGHM /// IGHV4-31 /// LOC100126583 /// LOC100134331 /// LOC642131 /// LOC652128 /// VSIG6, LOC652494, IGHV7-81 /// LOC100133739 /// LOC390530 /// LOC642131, IGH@ /// IGHA1 /// IGHA2 /// IGHV3OR16-13 /// LOC100126583, IGH@ /// IGHD /// IGHG1 /// LOC100126583 /// LOC100134331 /// LOC652128, IGH@ /// IGHG1 /// IGHG2 /// IGHM /// IGHV4-31, IGHA1 /// IGHD /// IGHG1 /// IGHG3 /// IGHM /// IGHV4-31 /// IGHV@ /// LOC100126583, IGHA1 /// IGHD /// IGHG1 /// IGHM /// IGHV4-31 /// IGHV@, IGHA1 /// IGHG1 /// IGHG3 /// IGHM /// IGHV4-31 /// IGHV@ /// LOC100133739, IGHA1 /// IGHG1 /// IGHG3 /// IGHM /// LOC652494, IGHA1 /// IGHG1 /// IGHV@, IGHA1 /// IGHG1 /// LOC100133862, IGHM, LOC100133862, LOC652494, IGJ, IGK@ /// IGKC, IGK@ /// IGKC /// IGKV1-5 /// LOC647506 /// LOC652694, IGK@ /// IGKC /// IGKV3-20 /// IGKV3D-11 /// IGKV3D-15 /// LOC440871, IGK@ /// IGKC /// LOC647506 /// LOC650405 /// LOC652493, IGK@ /// IGKV3-20 /// IGKV3D-11 /// IGKV3D-15 /// LOC440871, IGKC, IGKV1-5 /// LOC100130100 /// LOC647506 /// LOC650405 /// LOC652493 /// LOC652694, IGKC /// IGKV1-5 /// LOC647506 /// LOC652694, IGKV4-1, IGL@, IGLC1 /// IGLV2-11 /// IGLV2-18 /// IGLV2-23, IGL@ /// IGLC2 /// IGLV2-14, IGL@ /// IGLV1-36 /// IGLV1-44, IGL@ /// IGLV3-19, RPL14, IGLJ3, IGLL3, IGLV2-11 /// IGLV2-18 /// IGLV2-23, IGLV3-19, LOC100130100, LOC100132941 /// LOC647187 /// LOC647224, LOC339562, LOC652493, LOC91316, MGC29506, POU2AF1
8	36	Immune response	2.74E-17	CCL5, CCR7, CD2, CD247, CD27, CD3D, CD3E, CD6, CD8A, CD96, CST7, CTSW, CYTIP, FAIM3, GVIN1, GZMA, GZMB, GZMH, GZMK, IL12RB1, IL2RB, IL7R, KLRC4 /// KLRK1, KLRK1, LCK, NKG7, SAMD3, SIRPG, SLAMF7, TIGIT, TRA@ /// TRACTRA@ /// TRAC /// TRAJ17 /// TRAV20, TRAC, TRAT1, TRBC1, TRBC1 /// TRBC2
9	27	No significantly enriched BF	n.a.	AZIN1, C8orf38, C8orf59, COPS5, CPNE3, EBAG9, EIF3H, ENY2, IMPA1, INTS8, MED30, MRPL13, MRPL15, MRPS28, MTERFD1, NUDCD1, POLR2K, PXMP3, RAD21, SLC25A32, TATDN1, TCEB1, TMEM70, UBE2V2, WDSOF1, WDYHV1, YWHAZ
10	22	Antigen processing and presentation	2.38E-38	APOL6, B2M, CD74, GBP1, HCP5, HLA-A, HLA-A /// HLA-A29.1 /// HLA-B /// HLA-G /// HLA-H /// HLA-J HLA-B, HLA-B /// MICA HLA-C, HLA-DRB1 /// HLA-DRB2 /// HLA-DRB3 /// HLA-DRB4 /// HLA-DRB5 /// LOC100133484 /// LOC100133661 /// LOC100133811 /// LOC730415 /// RNASE2 /// ZNF749, HLA-E, HLA-F, HLA-G, IRF1, NLRC5, PSMB8, PSMB9, RARRES3, TAP1, TAP2, TAPBP
[illegible]	[illegible]	[illegible]	[illegible]	[illegible]

18	10	Cellular respiration	1.80E-16	ATP5J, ATP5O, COX7B, NDUFB3, COX5B, NDUFAB1, MDH1, ATP5G3, UQCRH, UQCRC2

Gene symbols highlighted in red were tested using HR assay and centrosome assay in the manuscript.
