# Supplementary material for: Weighted Frequent Gene Co-expression Network Mining to Identify Genes Involved in Genome Stability
Source: PLoS Comput Biol. 2012 Aug 30;8(8):e1002656. doi: 10.1371/journal.pcbi.1002656 (PMC3431293; doi:10.1371/journal.pcbi.1002656)
Supplement: Table S2 — Merged networks identified from normal tissue microarray datasets (β = 0.8, γ = 0.7, λ = 2.0, t = 1.0). (PDF) [file pcbi.1002656.s005.pdf]

Table S2: Merged networks identified from normal tissue microarray datasets (β=0.8, γ=0.7, λ=2.0, t=1.0)

Network ID	Network size in merged network	Top biological functions in the merged network	p-value	Gene member
1	198	Cellular respiration	5.31E-72	ACP1, ALDOA, ARMCX6 /// LOC653354, ATP5A1, ATP5E, ATP5F1, ATP5G1, ATP5J, ATP5J2, ATP5L, ATP5O, TP6V1E1, ATPAF1, AURKAIP1, BCAS2, BOLA3, BRP44, C14orf119, C14orf156, C14orf2, C15orf24, C16orf61, C18orf32, C19orf53, C19orf62, C19orf70, C1D, C1orf151, C1orf31, C1orf43, C20orf24, C20orf7, C2orf47, C6orf125, C6orf35 /// LOC730074, C7orf44, C8orf38, C9orf123, CAPZA1, CCDC56, CDC123, CHCHD2, CHCHD3, CHMP5, CIAPIN1, CISD2, CLTA, COMMD1, COPS2, COPS3, COPS4, COPS8, COX16, COX4I1, COX5A, COX5B, COX6A1, COX6B1, COX6C, COX7A2L, COX7B, COX7C, COX8A, CYC1, DBI, DDX1, EAPP, ECHS1, EIF3K, EIF4E2, EIF6, ETFA, EXOSC1, FDX1, FUT5 /// NDUFA11, GHITM, GLRX5, GSTK1, GSTO1, GUK1, HAX1, HINT1, IARS, IDE, IDH3A, IDH3B, ISCA2, KIAA0391 /// PSMA6, LOC152217, LOC727762 /// NDUFB4 LSM1, LSMD1, MAPKSP1, MCTS1, MDH1, MED7, METTL11A, METTL5, MRP63, MRPL11, MRPL13, MRPL15, MRPL21, MRPL22, MRPL4, MRPL47, MRPL51, MRPS12, MRPS17, MRPS18C, MRPS23, MRPS24, MRPS28, MRPS7, NDUFA1, NDUFA12, NDUFA13, NDUFA2, NDUFA3, NDUFA4, NDUFA6, NDUFA7, NDUFA8, NDUFA9, NDUFAB1, NDUFB1, NDUFB10, NDUFB2, NDUFB3, NDUFB5, NDUFB8 /// SEC31B, NDUFB9, NDUFC2, NDUFS3, NDUFS7, NDUFV1, NDUFV2, NFU1, NHP2L1, OAZ1, PARK7, PDHX, PDZD11, PFKM, POLR2K, POMP, POP4, PRKAG1, PSMA1, PSMB2, PSMB3, PSMB5, PSMB6, PSMD8, PXMP3, RABAC1, RABEPK, RPA3, RPL26L1, RRAGA, RTN4, SAP18, SDHD, SF3B14, SKP1, SLC25A11, SLC25A3, SLC25A4, SNRPC, SNX2, SNX3, SPCS1, STOML2, SUCLA2, SUCLG1, SUPT7L, TATDN1, TIMM17A, TIMM23, TMED3, TMEM126A, TMEM126B, TMEM85, TXNDC9, TXNL4A, UBE2D2, UBL7, UCRC, UQCR, UQCRC2, UQCRFS1, UQCRH, UQCRQ, USMG5, VDAC2, VPS29, ZBTB8OS, ZNHIT3
2	71	Protein synthesis	2.84E-99	ATP5G2, C6orf48, COMMD6, COX6A1, EIF3E, EIF3F, EIF3G, EIF3L, FAU, HMGN2, LOC100128936 /// RPL10A, LOC100130553 /// RPS18, LOC100131713 /// RPL29/// RPL29P4, LOC388474 /// LOC401640 /// LOC441034 /// LOC644029 /// LOC728139 /// LOC728179 /// RPL7A /// RPL7AP11, LOC441533, LOC649299 /// RPL36A, NACA, NME1 /// NME1-NME2 /// NME2, PFDN5, POLR2I, RPL10, RPL10A, RPL12, RPL13A, RPL14 /// RPL14L, RPL17, RPL18, RPL19, RPL23, RPL23A, RPL24, RPL24 /// SLC36A2, RPL29 /// RPL29P4, RPL30, RPL31, RPL32, RPL35, RPL35A, RPL36A, RPL38, RPL41, RPL7, RPL7A, RPL9, RPLP1,

2	71	Protein synthesis	2.84E-99	ATP5G2, C6orf48, COMMD6, COX6A1, EIF3E, EIF3F, EIF3G, EIF3L, FAU, HMGN2, LOC100128936 /// RPL10A, LOC100130553 /// RPS18, LOC100131713 /// RPL29/// RPL29P4, LOC388474 /// LOC401640 /// LOC441034 /// LOC644029 /// LOC728139 /// LOC728179 /// RPL7A /// RPL7AP11, LOC441533, LOC649299 /// RPL36A, NACA, NME1 /// NME1-NME2 /// NME2, PFDN5, POLR2I, RPL10, RPL10A, RPL12, RPL13A, RPL14 /// RPL14L, RPL17, RPL18, RPL19, RPL23, RPL23A, RPL24, RPL24 /// SLC36A2, RPL29 /// RPL29P4, RPL30, RPL31, RPL32, RPL35, RPL35A, RPL36A, RPL38, RPL41, RPL7, RPL7A, RPL9, RPLP1, RPLP2, RPS10, RPS10L, RPS11, RPS12, RPS13, RPS14, RPS15, RPS15A, RPS16, RPS17, RPS19, RPS2, RPS20, RPS21, RPS24, RPS27, RPS29, RPS5, RPS6, RPS9, TCEB2, UBA52, UXT, hCG_16001 /// RPL23A, hCG_21078 /// RPL27A
3	60	No significantly enriched BF	n.a.	ADAM33, ADH4, ALMS1, ATF7IP, C9orf64, CCDC152, CDC2L5, CEP27, CMBL, DBT, DDX59, DLGAP4, FAM161B, FAM63A, FBXW12, FLJ12151, GTF2H3, GTSE1, HCG2P7, HSD17B7, KIAA0894, KIAA1245 /// NBPF1 /// NBPF10 /// NBPF11 /// NBPF14 /// NBPF16 /// NBPF20 /// NBPF3 /// NBPF8 /// NBPF9 /// RP11-94I2.2, LOC100128510, LOC100132134 /// LOC100134401 /// LOC653188, LOC100132247 /// LOC23117 /// LOC613037, LOC100132832 /// PMS2L1 /// PMS2L2, LOC152719, LOC23117 /// LOC642799 /// LOC729602, LOC340085, LOC440354 /// LOC595101 /// LOC641298 /// LOC728423 /// LOC729513 /// SMG1, LOC441258, LOC493754, LOC51057, LOC641298, LOC727820, LOC728153, LOC728678, LRRFIP1, MUTED, NKTR, NLN, NUMBL, ORAI2, PDE4C, PECR, PGF, PRR11, RANBP2 /// RGPD1 /// RGPD2 /// RGPD3 /// RGPD4 /// RGPD5 /// RGPD6 /// RGPD7 /// RGPD8, RP5-886K2.1, SLC22A3, SLC35E1, UBXN2A, XRCC2, ZC3H7B, ZNF160, ZNF44, ZNF587, ZNF611, chromosome 1 open reading frame
