# Supplementary material for: Weighted Frequent Gene Co-expression Network Mining to Identify Genes Involved in Genome Stability
Source: PLoS Comput Biol. 2012 Aug 30;8(8):e1002656. doi: 10.1371/journal.pcbi.1002656 (PMC3431293; doi:10.1371/journal.pcbi.1002656)
Supplement: Table S3 — Comparison of the top three major networks identified from cancer vs. normal tissue microarray datasets using different parameter settings. (PDF) [file pcbi.1002656.s006.pdf]

Table S3: Comparison of the top three major networks identified from Cancer vs. normal tissue microarray datasets using different parameter settings.

QCM parameter setting	Networks from cancer datasets	Networks from normal tissue datasets	Common genes shared by top networks from cancer and normal datasets
$\beta = 0.5, \gamma = 0.6$	Size: 670, BF: cell cycle	Size: 152, BF: cellular respiration	43
	Size: 603, BF: immune response	Size: 147, BF: protein synthesis	
	Size: 555, BF: cell cycle	Size: 126, BF: Cellular respiration	
$\beta = 0.5, \gamma = 0.7$	Size: 481, BF: cell cycle	Size: 152, BF: cellular respiration	27
	Size: 381, BF: cell cycle	Size: 147, BF: protein synthesis	
	Size: 333, BF: immune response	Size: 126, BF: Cellular respiration	
$\beta = 0.5, \gamma = 0.8$	Size: 462, BF: cell cycle	Size: 75, BF: Cellular respiration	66
	Size: 191, BF: immune response	Size: 70, BF: unknown	
	Size: 144, BF: protein synthesis	Size: 69, BF: protein synthesis	
$\beta = 0.8, \gamma = 0.7$	*Size: 667, BF: cell cycle	*Size: 198, BF: cellular respiration	84
	*Size: 442, BF: immune response	*Size: 71, BF: protein synthesis	
	*Size: 171, BF: protein synthesis	*Size: 60, BF: unknown	
$\beta = 0.9, \gamma = 0.7$	*Size: 654, BF: cell cycle	*Size: 173, BF: cellular respiration	73
	*Size: 429, BF: immune response	*Size: 68, BF: protein synthesis	
	*Size: 160, BF: protein synthesis	*Size: 59, BF: unknown	
$\beta = 0.9, \gamma = 0.8$	*Size: 399, BF: cell cycle	*Size: 30, BF: unknown	0
	*Size: 266, BF: immune response	*Size: 19, BF: cellular respiration	
	*Size: 134, BF: protein synthesis		

*size is for the networks after merging step. BF: biological function. The above table does not include the results from $\beta = 0.8, \gamma = 0.8$, which were shown in the manuscript.
