# Supplementary material for: Weighted Frequent Gene Co-expression Network Mining to Identify Genes Involved in Genome Stability
Source: PLoS Comput Biol. 2012 Aug 30;8(8):e1002656. doi: 10.1371/journal.pcbi.1002656 (PMC3431293; doi:10.1371/journal.pcbi.1002656)
Supplement: Table S4 — Details of the networks identified from lung cancer microarray datasets using different parameter settings. (PDF) [file pcbi.1002656.s007.pdf]

Table S4: Details of the networks identified from lung cancer microarray datasets using different parameter settings. Size is for the networks after merging step. BF: biological function.

QCM parameter setting	Networks from lung cancer datasets	Network Member
β = 0.8, γ = 0.99	Size: 54, BF: Mismatch repair	ADAMTS17, ALB, ALDH1A2, ATP1A2, B3GAT2, BOC, C20orf186, C8orf22, CHRNA1, COL2A1, CYTL1, DLK1, DPP6, EDN3, EYA1, FAT3, FSTL5, GABRA2, GAS2, GCG, HOTAIR, ITGA10, KCNJ4, LHX8, LOC100131819, LOC100132832 /// LOC441259 /// LOC730324 /// PMS2 /// PMS2L1 /// PMS2L12 /// PMS2L2 /// PMS2L3 /// PMS2L5, LOC100134259, LOC100192378, LOC143381, LOC257152, LOC283867, MAGEL2, MEOX2, MKX, MMP16, MYH8, NPTX2, OLFM3, PHOX2B, PRG4, PRSS35, RASL11B, RBM24, RUNX1T1, SCRG1, SIX3, STMN2, TAC1, THBS4, TIMP4, TNMD, TRO, ZFHX4, ZNF804A
	Size: 21, BF: Immune response	DEFA5, GP2, INHA, PDIA2, KIR2DS1 /// KIR2DS2 /// KIR2DS4 NEUROD1, KIR2DL5A, PLCXD3, CRYBA2, PAH, RFX6, KIR2DS5, KIR3DL3, INSM1, KLK12, KIR3DL1, ADAM6, HEPACAM2, KIR2DS1, SNTA1, PLUNC
	Size: 17, BF: ECM organization	PANX3,RNF175, SP7, ACAN, IBS, SNORD114-3, ANGPTL1, INSC, PNLDC1, C1QTNF2, SCARF2, KCNMB2, C1QTNF7, AMBN, EMX2, EMID1, HOXA9
	Size: 17, BF: No Sig. enriched BF	MTMR7, OBP2A, B3GALT2, OBP2B, ASCL1, CALCA, IP6K3, SPP2, BPIL1, SLC14A2, ST18 , P11-167P23.2, CNGA3, CA6, DDC, FLJ38379, C20orf56
	Size: 16, BF: No Sig. enriched BF	INSL4, LOC285986, ADH6, CRISP2, RELN, ASB4, SPINK4, MTTP, AMBP, FGL1, C8A, BMP6, PGC, PLA2G10, MUC13, RETNLB
	Size: 16, BF: mRNA modification	APOBEC1, FLJ35767, C19orf30, B3GNT6, DIRAS3, ACRC, AREGB, APOBEC2, COX8C, SERPIND1, C16orf73, WDR69, ZNF560, CPNE4, PCDHGA4, ISL1
	Size: 13, BF: No Sig. enriched BF	ASPRV1, SPINK7, TGM3, SCG3, CHGA, RUNDC3A, INA, PRMT8, IGFL1, FLG, KRT24, LOC643008, POU4F2
	Size: 13, BF: epidermis development	FLG2, KRT34, SPRR2G, WFDC12, CDSN, KRT2, RPTN, ARG1, HESRG, C1orf186, LMO1, BPIL2, LOC284561
	Size: 12, BF: No Sig. enriched BF	APLN, BCAR4, CYP46A1, SLC6A1, ROBO4, TM4SF18, ANGPT2, PRND, ELTD1, NR5A2, C18orf34, CETP
	Size: 11, BF: No Sig.	ALDH8A1, SMPX, MYOD1, DMRT1, CDKAL1,

	enriched BF	FBXO27, XK, ZNF599, SHH, FGFBP3, OSBPL11
	Size: 10, BF: Hormone activity	C5orf17, KRT81, CGA, SPINK6, SH3GL2, CCK, FLJ37786, PTPRN, BMY2FP, MARCH11
β = 0.8, γ = 0.95	Size: 105, BF: Mismatch repair	ADAMTS17, ALB, ALDH1A2, ATP1A2, B3GAT2, BOC, C1QTNF4, C20orf186, C8orf22, CADPS, CCDC28B, CCDC8, CCDC88A, CHRNA1, COL2A1, CRISPLD1, CYTL1, DAZ1 /// DAZ2 /// DAZ3 /// DAZ4 /// LOC732447, DIO3, DLK1, DLX4, DNHD2, DPP6, DPY19L2P2, DUSP2, EDN3, ELAVL4, EN1, EYA1, EYA4, FAM124A, FAT3, FBXO10, FOXR2, FSTL5, GABRA2, GAS2, GCG, GPER, GPR20, HOTAIR, HOXA11, IGF2BP1, IGLON5, IL11RA, IL17RD, ITGA10, KCNJ4, KIAA1024, LHX8, LOC100127983, LOC100128098, LOC100131819, LOC100132832 /// LOC441259 /// LOC730324 /// PMS2 /// PMS2L1 /// PMS2L12 /// PMS2L2 /// PMS2L3 /// PMS2L5, LOC100134259, LOC100192378, LOC143381, LOC257152, LOC283867, LOC654342, LOC729970, LRRN3, MAGEL2, MEOX2, MFAP3L, MKX, MMP16, MYH8, NKX2-2, NPTX2, ODZ3, OLFM3, PDE7B, PEG3, PHOX2B, PRG4, PRSS35, PTGFR, RASL11B, RBM24, RIMS2, RUNX1T1, SCRG1, SEMA3E, SIX3, SNAP91, STMN2, SYDE2, TAC1, TCEAL2, TCEAL7, THBS4, TIMP4, TNMD, TRIM63, TRO, TTC28, UNC5C, VAT1L, ZFHX4, ZIC1, ZNF804A
	Size: 50, BF: ECM construction	ACAN, ADAM22, AMACR /// C1QTNF3, AMBN, ANGPTL1, C1QTNF2, C1QTNF7, CCDC3, COL8A2, CPEB1, CPXM1, DACT3, DCHS1, DLX5, DLX6, ELN, EMID1, EMX2, FBXL7, FLJ34048, FLRT1, HAPLN1, HOXA9, IBSP, IGDCC4, IL17D, INSC, KCNMB2, LOC100128178, LOC100130725 /// PAGE2 /// PAGE2B, LOC100133471 /// LOC285103, LRRC15, MAB21L1, MST075 /// RPA1, MSX1, NCAM1, NPY, OGN, PANX3, PCOLCE, PNLDC1, PTH1R, RNF175, SATB2, SCARF2, SNORD114-3, SP7, ZNF423, ZNF521, ZNF575
	Size: 35, BF: Immune response	ADAM6, AGXT2L1, CHGB, CHRNA9, CRYBA2, DEFA5, GDF10, GP2, HEPACAM2, INHA, INSM1, KIR2DL3, KIR2DL5A, KIR2DS1, KIR2DS1 /// KIR2DS2 /// KIR2DS4, KIR2DS3, KIR2DS5, KIR3DL1, KIR3DL1 /// KIR3DS1, KIR3DL2 /// LOC727787, KIR3DL3, KLK12, NEUROD1, PAGE1, PAGE4, PAH, PDIA2, PLCXD3, PLUNC, PPARGC1A, RET, RFX6, SNTA1, TRIM72, TRPM5
	Size: 34, BF: Immune response	INSL4, LOC285986, ADH6, CRISP2, RELN, ASB4, SPINK4, MTTP, AMBP, FGL1, C8A, BMP6, PGC, PLA2G10, SLC26A4, ARSE, TESC, MUC13, RETNLB, FOXA3, OLFM4, RNF183, AKR1C4, MUC5B,

		LOC100132502 /// NCRNA00166, MUC3A, GOLT1A, SPINK1, ACHE, ABCC9, C1orf225, CREB3L1, PBLD, CATSPERB,
	Size: 29, BF: No Sig. enriched BF	AIF1, APBB1IP, C1QA, C1QB, C1QC, CD14, CYBB, FCER1G, FCGR1A /// FCGR1C, FCGR1B, FERMT3, FPR3, GPR84, HAVCR2, HK3, IL21R, IL4I1, ITGAM, ITGB2, KMO, LAIR1, LAPTM5, LILRB1, NCKAP1L, SIGLEC10, SLC7A7, TFEC, TNFAIP8L2, VSIG4
	Size: 26, BF: Epidermis development	ASPRV1, SPINK7, TGM3, SCG3, CHGA, DSC1, DSG1, C9orf169 /// LOC100130547, RUNDC3A, INA, PRMT8, IGFL1, FLG, KRT24, LOC643008, TKTL1, POU4F2, CD177, CNFN, NEFM, KLK13, PRSS27, TGM1, APOBEC3A, SLC39A2, P2RY1
	Size: 26, BF: No Sig. enriched BF	ASCL1, B3GALT2, BPIL1, C10orf108, C20orf56, C9orf135, CA6, CALCA, CNGA3, DDC, DUSP13, FLJ38379, GMPR, IL1RL1, IP6K3, IYD, MTMR7, OBP2A, OBP2A /// OBP2B, OBP2B, PTPRN2, RP11-167P23.2, SFTA1P, SLC14A2, SPP2, ST18
	Size: 25, BF: mRNA modification	ACRC, APOBEC1, APOBEC2, AREG /// AREGB, AREGB, B3GNT6, C14orf39, C16orf73, C19orf30, CIB3, COX8C, CPNE4, DIRAS3, FLJ30851 /// LOC375190, FLJ35767, GCK, HOXD3, ISL1, PCDH8, PCDHGA4, SERPIND1, WDR69, ZFP42, ZNF560, tcag7.1188
	Size: 22, BF: No Sig. enriched BF	ARMC3, C1orf173, C1orf194, C1orf87, C20orf85, C9orf24, CAPSL, CASC1, DYNLRB2, FAM81B, LOC643037, MS4A8B, OMG, RSPH1, S100A1L, SPAG6, TEKT1, TMEM146, TMEM190, TTC29, WDR38, ZBBX
	Size: 22, BF: No Sig. enriched BF	C5orf17, KRT81, CGA, SPINK6, SH3GL2, KRT86 /// LOC100134394, CCK, FLJ37786, PTPRN, RBMY2FP, MARCH11, CASP14, HCG4, SMOC1, C10orf82, CDH12, ASTN1, GPR50, TMEM91, ELAVL2, KLK5, C1QL1
	Size: 20, BF: No Sig. enriched BF	ANGPT2, APLN, BCAR4, C18orf34, CD93, CDH5, CETP, CYP46A1, ECSM2, ELTD1, ESM1, HGF, LOC100132810 /// LOC441179, LYVE1, NR5A2, PRND, ROBO4, SLC6A1, TM4SF18, VWF
	Size: 19, BF: No Sig. enriched BF	ACTL8, ADH1A, ADH1C, CRISP3, CT47.7 /// CT47.8 /// RP6-166C19.1 /// RP6-166C19.10 /// RP6-166C19.11 /// RP6-166C19.2 /// RP6-166C19.3 /// RP6-166C19.4 /// RP6-166C19.5 /// RP6-166C19.6 /// RP6-166C19.9, DSCR8, LOC100130648 /// SSX2 /// SSX2B, LOC441601, SAGE1, SERPINB10, SPANXA1 /// SPANXA2 /// SPANXB1 /// SPANXB2 /// SPANXC /// SPANXF1, SPANXA1 /// SPANXA2 /// SPANXC, SPANXB1 /// SPANXB2 /// SPANXF1, SPANXC, TNNT3, TPTE, TSPYL6, WDR21B, WDR21C

	Size: 16, BF: No Sig. enriched BF	GUSBL2 /// LOC728411, SLC6A15, THAP9, MRPL1, GDNF, CSN1S1, SULT1E1, SDAD1, EPHA7, NUP54, LETM2, PAX1, KCNC1, ALK, KIAA2022, WNK4
	Size: 16, BF: No Sig. enriched BF	CDC2L5, NLN, DDX59, PGF, SLC35E1, CCDC152, C9orf64, CMBL, FAM63A, LOC152719, DIP2A, ZNF160, FAM161B, PDE4C, DBT, ATF7IP
	Size: 16, BF: T-cell activation	DOCK8, PTPRC, CD53, EVI2B, ARHGAP30, SASH3, IL10RA, APBB1IP, CYTH4, FERMT3, BIN2, DOCK2, CD48, NCKAP1L, SELPLG, TFEC

Only networks with size above 15 are shown here.
