# Supplementary material for: Weighted Frequent Gene Co-expression Network Mining to Identify Genes Involved in Genome Stability
Source: PLoS Comput Biol. 2012 Aug 30;8(8):e1002656. doi: 10.1371/journal.pcbi.1002656 (PMC3431293; doi:10.1371/journal.pcbi.1002656)
Supplement: Table S5 — Details of the networks identified from normal lung tissue microarray datasets using different parameter settings. (PDF) [file pcbi.1002656.s008.pdf]

Table S5: Details of the networks identified from normal lung tissue microarray datasets using different parameter settings. Size is for the networks after merging step. BF: biological function.

QCM parameter setting	Networks from lung cancer datasets	Network Member
β = 0.8, γ = 0.99	Size: 14, BF: No Sig. Enriched BF	C9orf24, MS4A8B, TMEM146, TEKT2, FAM81B, ARMC3, C20orf85, C9orf116,DYNLRB2, LRRC23, CAPS, ROPN1L, C11orf70, C6orf206
	Size: 12, BF: Immune response	IGH@ /// IGHA1 /// IGHA2 /// IGHG1 /// IGHG3 /// IGHM /// IGHV4-31 /// IGHV7-81 /// LOC100133739 /// LOC390530 /// LOC642131, IGHM /// LOC100133862,IGH@ /// IGHA1 /// IGHA2 /// IGHG1 /// IGHG2 /// IGHG3 /// IGHM /// IGHV4-31 /// LOC100126583 /// LOC100133739 /// LOC652494,IGHA1 /// IGHG1 /// IGHG3 /// IGHM /// IGHV4-31 /// IGHV@ /// LOC100133739, IGHA1 /// IGHD /// IGHG1 /// IGHG3 /// IGHM /// IGHV4-31 /// IGHV@ /// LOC100126583, IGHA1 /// IGHG1 /// IGHV@, IGHA1 /// IGHG1 /// LOC100133862, IGH@ /// IGHA1 /// IGHA2 /// IGHD /// IGHG1 /// IGHG3 /// IGHG4 /// IGHM /// IGHV4-31 /// LOC100126583 /// LOC100133739 /// LOC100134331 /// LOC642131 /// LOC652128 /// VSIG6, IGH@ /// IGHA1 /// IGHA2 /// IGHG1 /// IGHG3 /// IGHM /// IGHV4-31 /// IGHV@ /// LOC100133739,IGHA1 /// IGHD /// IGHG1 /// IGHM /// IGHV4-31 /// IGHV@, IGHM /// LOC652494, IGLJ3
β = 0.8, γ = 0.95	Size: 71, BF: Microtubule based process	AKAP14, ARMC3, C10orf63, C11orf70, C11orf88, C14orf45, C19orf51, C1orf192, C1orf87, C20orf85, C2orf39, C6, C6orf165, C6orf206, C7orf57, C9orf116, C9orf24, CAPS, CAPSL, CASC1, CCDC11, CCDC113, CCDC114, CCDC146, CCDC19, CCDC39, CCDC65, CCDC74A /// CCDC74B, CCDC78, CETN2, DNAH2, DNAI1, DNAI2, DNHD2, DYDC2, DYNLRB2, EFCAB1, FABP6, FAM81B, HYDIN /// HYDIN2 /// LOC100133820, IQCG, LOC643037, LRRC23, LRRC34, LRRC46, MDH1B, MNS1, MS4A8B, NME5, ROPN1L, RSHL3, RSPH1, SOX2, SPATA17, SPATA18, SPATA4, STK33, TEKT1, TEKT2, TMEM146, TMEM190, TSPAN1, TTC25, TTC29, TUBA4B, VWA3B, WDR63, WDR66, WDR69, WDR78, ZMYND10
	Size: 36, BF: Respiratory electron transport chain	ANAPC11 /// LOC100131844, ATP5J2, BOLA3, C11orf1, C14orf2, C17orf37, C17orf61, C19orf53, C1orf151, C2orf79, CCDC53, COX16, COX5B, COX6B1, COX7A2, COX7C, COX8A, HIGD2A, LOC729776, MEA1, MRP63, MRPL33, MRPL40, MYEOV2, NDUFA1, NDUFA11, NDUFA2, NDUFA3, NDUFB1, PDZD11, RWDD1, TAF10, TIMM8B, TMEM134, UQCR, USMG5

	Size: 19, BF: protein synthesis	DKC1, WDR3, NOP16, PNO1, BYSL, CD3EAP, SERPINB2, CCDC86, KIAA0020, MAK16, POP1, RRS1, URB2, MRTO4, WDSOF1, GPATCH4, C10orf2, PUS1, PNPT1
	Size: 15, BF: Immune response	IGH@ /// IGHA1 /// IGHA2 /// IGHG1 /// IGHG3 /// IGHM /// IGHV4-31 /// IGHV7-81 /// LOC100133739 /// LOC390530 /// LOC642131, IGHM /// LOC100133862, IGH@ /// IGHA1 /// IGHA2 /// IGHG1 /// IGHG2 /// IGHG3 /// IGHM /// IGHV4-31 /// LOC100126583 /// LOC100133739 /// LOC652494, IGHA1 /// IGHG1 /// IGHG3 /// IGHM /// IGHV4-31 /// IGHV@ /// LOC100133739, IGHA1 /// IGHD /// IGHG1 /// IGHG3 /// IGHM /// IGHV4-31 /// IGHV@ /// LOC100126583, IGHG1, IGHA1 /// IGHG1 /// IGHV@, IGHA1 /// IGHG1 /// LOC100133862, IGH@ /// IGHA1 /// IGHA2 /// IGHD /// IGHG1 /// IGHG3 /// IGHG4 /// IGHM /// IGHV4-31 /// LOC100126583 /// LOC100133739 /// LOC100134331 /// LOC642131 /// LOC652128 /// VSIG6, IGH@ /// IGHA1 /// IGHA2 /// IGHG1 /// IGHG3 /// IGHM /// IGHV4-31 /// IGHV@ /// LOC100133739, IGHA1 /// IGHD /// IGHG1 /// IGHM /// IGHV4-31 /// IGHV@, IGHM /// LOC652494, IGH@ /// IGHA1 /// IGHA2 /// IGHD /// IGHG1 /// IGHG3 /// IGHG4 /// IGHM /// IGHV4-31 /// LOC100126583 /// LOC100134331 /// LOC642131 /// LOC652128 /// VSIG6, IGK@ /// IGKV3-20 /// IGKV3D-11 /// IGKV3D-15 /// LOC440871, IGLJ3
	Size: 14, BF: NO Sig. enriched BF	PDE4C, PRR11, NLN, CDC2L5, FLJ12151, CCDC152, PGF, SLC22A3, CEP27, LOC152719, C9orf64, DLGAP4, DBT, SLC35E1
	Size: 13, BF: Immune response	LOC339562, LOC652493, IGKC /// IGKV1-5 /// LOC647506 /// LOC652694, IGKC /// IGKV1-5 /// LOC100130100 /// LOC647506 /// LOC650405 /// LOC652493 /// LOC652694, IGK@ /// IGKC /// LOC647506 /// LOC650405 /// LOC652493, LOC100130100, IGK@ /// IGKV3-20 /// IGKV3D-11 /// IGKV3D-15 /// LOC440871, IGL@ /// IGLC1 /// IGLV2-11 /// IGLV2-18 /// IGLV2-23, IGLV2-11 /// IGLV2-18 /// IGLV2-23, IGLJ3, IGK@ /// IGKC /// IGKV3-20 /// IGKV3D-11 /// IGKV3D-15 /// LOC440871, IGK@ /// IGKC /// IGKV1-5 /// LOC647506 /// LOC652694, IGL@ /// IGLV1-36 /// IGLV1-44
	Size: 11, BF: Sulfur compound metabolic process	C1orf173, CHST9, PROM1, C10orf81, SLC27A2, FAM154B, C8orf47, C4orf22, GSTA3, CHST6, IQCD
	Size: 11, BF: Protein synthesis	LOC100131713 /// RPL29 /// RPL29P4, RPL29 /// RPL29P4, RPL31, RPL38, RPL37, RPS15A, RPS29, SNRPD2, RPL32, RPS13, RPS10

	Size: 10, BF: Cellular component assembly	SPTBN1, TJP1, TNS1, ADARB1, MYH10, ANO6, KANK2, LIMCH1, SLIT2, TMEM47
