# Supplementary material for: Weighted Frequent Gene Co-expression Network Mining to Identify Genes Involved in Genome Stability
Source: PLoS Comput Biol. 2012 Aug 30;8(8):e1002656. doi: 10.1371/journal.pcbi.1002656 (PMC3431293; doi:10.1371/journal.pcbi.1002656)
Supplement: Table S6 — The sequences of siRNA probes used for target gene depletion in HeLa and Hs578T cell lines. (PDF) [file pcbi.1002656.s009.pdf]

Table S6: The sequences of siRNA probes used for target gene depletion in HeLa and Hs578T cell lines.

Protein	siRNA 1	siRNA 2	siRNA 3
GL2	CGUACGCGGAAUACUUCGA		
	UCGAAGUAUUCCGCGUACG		
BRCA1	ACUGAAGAGUGAGAGGAGC		
	GCUCCUCUCACUCUUCAGU		
BARD1	CAGUAACAUGUCCGAUGAA		
	UUCAUCGGACAUGUUACUG		
ASF1B	UGGCUCGGCUGAGAGUGAGGAAUUU	GCGGACGACCUGGAGUGGAAGAUCA	UCAUCACCUGCACCUACCAUGGACA
	AAAUUCCUCACUCUCAGCCGAGCCA	UGAUCUUCCACUCCAGGUCGUCCGC	UGUCCAUGGUAGGUGCAGGUGAUGA
ASPM	GCACAGUCCAGUUUGACCUUCAUAA	GCGCUGUUUCGUGUUGCAGCCUAAA	GGAGAGAGAGAAAGCUGCAAGAAUU
	UUAUGAAGGUCAAACUGGACUGUGC	UUUAGGCUGCAACACGAAACAGCGC	AAUUCUUGCAGCUUUCUCUCUCUCC
CDCA3	UACUUCACUCAGCUGUUUCACCAGU	UUCCUCAACAGAUAACUGGGUACCC	UUUCUGUGGGCUGUCUUGCUUCCUC
	ACUGGUGAAACAGCUGAGUGAAGUA	GGGUACCCAGUUAUCUGUUGAGGAA	GAGGAAGCAAGACAGCCCACAGAAA
KIF14	UAUACUUUCCAACUGUAGUUGUUCC	UUGAGAAGAAAGCUCUUGCUGAGCC	UUAACUUCUUGUCUCAACUCAUCCU
	GGAACAACUACAGUUGGAAAGUAUA	GGCUCAGCAAGAGCUUUCUUCUCAA	AGGAUGAGUUGAGACAAGAAGUUAA
KIF23	AAAUUUCGCAUGACGGCAAAGGUGG	UAUUCUGCAUCUCCAGCUGUUUGGC	UAUGGGAUCAAACGGCACCUCUUCC
	CCACCUUUGCCGUCAUGCGAAAUUU	GCCAAACAGCUGGAGAUGCAGAAUA	GGAAGAGGUGCCGUUUGAUCCCAUA
MKI67	CAGUGUUGCUCAGGGAACAACUAAU	UCUGGAAGACCUGACUGGCUUUAAA	GGAAGGUCUACAGAGUUCAGGAAUA
	AUUAGUUGUUCCCUGAGCAACACUG	UUUAAAGCCAGUCAGGUCUUCCAGA	UAUUCCUGAACUCUGUAGACCUUCC
NUSAP1	UAAUUUGUGUGUCCCAAGCACAGCC	UAUUGGAGACUGGAGUCUGCGUUGC	UUUCGUUCUUGCUCGCGUUUCUUCC
	GGCUGUGCUUGGGACACACAAAUUA	GCAACGCAGACUCCAGUCUCCAAUA	GGAAGAAACGCGAGCAAGAACGAAA
ZWINT	UGGCCUUCAGCUCUUUCCAUUGUUC	AUCUGAGUCAGGGCCUUGGUGAGGC	AGGCCUACAGGUUCCAAGAUGCCUG
	GAACAAUGGAAAGAGCUGAAGGCCA	GCCUCACCAAGGCCCUGACUCAGAU	CAGGCAUCUUGGAACCUGUAGGCCU
DLG7	UUUAAAGGAGUCCAGGUGUAACUGG	UAAACAAUCCACCUUCAAGUCUGUC	UUUACAAGAAAUACCCUUGUCGGGU
	CCAGUUACACCUGGACUCCUUUAAA	GACAGACUUGAAGGUGGAUUGUUUA	ACCCGACAAGGGUAUUUCUUGUAAA
BLM	GRODEN LAB GIFT		
